# Associations of time-weighted individual exposure to ambient particulate matter with carotid atherosclerosis in Beijing, China

**DOI:** 10.1186/s12940-023-00995-8

**Published:** 2023-05-29

**Authors:** Ze Han, Xiaoyu Zhao, Zongkai Xu, Jinqi Wang, Rui Jin, Yueruijing Liu, Zhiyuan Wu, Jie Zhang, Xia Li, Xiuhua Guo, Lixin Tao

**Affiliations:** 1 School of Public Health, Capital Medical University, No.10 Xitoutiao, You’anmenWai, Fengtai District, Beijing, 100069 China; 2 Beijing Municipal Key Laboratory of Clinical Epidemiology, Beijing, 100069 China; 3 Department of Public Health, School of Medical and Health Sciences, Edith Cowan University, Perth, Australia; 4 Department of Mathematics and Statistics, La Trobe University, Melbourne, 3086 Australia

**Keywords:** Carotid atherosclerosis, PM_2.5_, PM_10_, Quantile g-computation

## Abstract

**Background:**

Time-location information (time spent on commuting, indoors and outdoors around residential and work places and physical activity) and infiltrated outdoor pollution was less considered estimating individual exposure to ambient air pollution. Studies investigating the association between individual exposure to particulate matter (PM) with aerodynamic diameter < 10 μm (PM_10_) and < 2.5 μm (PM_2.5_) and carotid atherosclerosis presented inconsistent results. Moreover, combined effect of pollutants on carotid atherosclerosis was not fully explored. We aimed to investigate the association between long-term individual time-weighted average exposure to PM_2.5_ and PM_10_ and the risk of carotid atherosclerosis, and further explore the overall effect of co-exposure to pollutants on carotid atherosclerosis.

**Methods:**

The study population included 3069 participants derived from the Beijing Health Management Cohort (BHMC) study. Daily concentration of ambient air pollutants was estimated by land-use regression model at both residential and work addresses, and one- and two-year time-weighted average individual exposure was calculated by further considering personal activity pattern and infiltration of ambient air pollution indoors. We explored the association of PM_2.5_ and PM_10_ with carotid atherosclerosis and pooled the overall effect of co-exposure to ambient air pollutants by quantile g-computation.

**Results:**

A significant association between time-weighted average exposure to PM_2.5_ and PM_10_ and carotid atherosclerosis was observed. Per interquartile range increase in two-year exposure to PM_2.5_ (Hazard ratio (HR): 1.322, 95% confidence interval (CI): 1.219–1.434) and PM_10_ (HR:1.213, 95% CI: 1.116–1.319) showed the strongest association with carotid atherosclerosis, respectively. Individuals in higher quartiles of pollutants were at higher risk for carotid atherosclerosis compared with those in the lowest quartile group. Concentration response functions documented the nearly linear and nonlinear relationship and interpreted the upward trends of the risk for carotid atherosclerosis with increasing level of pollutant concentrations. Moreover, effect estimates for the mixture of pollutants and carotid atherosclerosis were larger than any of the individual pollutants (HR (95% CI) was 1.510 (1.338–1.704) and 1.613 (1.428–1.822) per quartile increase for one-year and two-year time-weighted average exposure, respectively).

**Conclusions:**

Individual time-weighted average exposure to PM_2.5_ and PM_10_ was associated with carotid atherosclerosis. Co-exposure to ambient air pollution was also positively associated with carotid atherosclerosis.

**Supplementary Information:**

The online version contains supplementary material available at 10.1186/s12940-023-00995-8.

## Introduction

Cardiovascular diseases (CVDs), the leading cause of death globally, lead to approximately 32% of global deaths (https://www.who.int/en/news-room/fact-sheets/detail/cardiovascular-diseases(cvds)). Atherosclerosis is the primary underlying pathology of CVDs [1]. The development of atherosclerosis is the result of total exposure to individual and environmental risk factors interacting with genetic susceptibility [2]. Ambient pollution has been identified as the most important environmental risk factor [3]. Ambient particulate matter with aerodynamic diameter < 10 μm (PM_10_) and < 2.5 μm (PM_2.5_) are considered to be the main adverse factor of human health [4] because of the broad range of toxic substances they contain. Exposure to ambient particulate matter (PM) would contribute to the progression of subclinical disease and the development of atherosclerosis [5, 6].

Carotid atherosclerosis is a reliable and well-developed clinical indicator of CVDs [7]. The progression would trigger 20% of the whole risk of ischemic stroke [8] and 50% deaths of atherosclerotic diseases [9]. Carotid intima-media thickness (CIMT) or carotid plaque are well-established quantitative measurements for carotid atherosclerosis and better biomarkers for predicting the risk of CVDs and atherosclerotic vascular disease [10]. Association between PM and atherosclerotic lesion has been explored by animal models and population-based studies [11, 12], but the results were not generally consistent. Long-term exposure to PM was demonstrated as an important risk factor for CIMT in both cross-sectional and longitudinal studies among late midlife women [13] or women transitioning through menopause [14], adolescents or young adults [15], children [16], general population [17, 18] and even HIV-positive adolescents [19]. But such association was not found in some other studies for either PM_2.5_ [20, 21] or PM_10_ [15]. PM_2.5_ and PM_10_ exposure was associated with the prevalence and progression of carotid plaque, but not with CIMT [22]. Besides, investigators reported that the significant association of PM_2.5_ and PM_10_ with CIMT or carotid plaque disappeared after adjusting for cardiovascular and socioeconomic risk factors [23].

Estimation of individual exposure to PM_2.5_ and PM_10_ simply based on monitoring stations would induce exposure misclassification [13, 21]. The significant association between PM_2.5_ and atherosclerosis disappeared after adjusting for metropolitan area [24], which interprets the implication of spatial characteristics for pollutant distribution. Land-use regression (LUR) model [25, 26] is a reliable method to estimate individual PM exposure considering both influencing factors and spatiotemporal characteristics of pollutant transmission. However, estimation from simple prediction model failed to account for individual activity characteristics and infiltrated ambient pollution. The Multi-Ethnic Study of Atherosclerosis and Air Pollution (MESA Air) study was well-performed with complete protocol to estimate long-term individual exposure to PM_2.5_ [27]. Time spent both indoors and outdoors and infiltrated ambient PM_2.5_ and PM_10_ was accounted for [21, 27]. But exposure on commuting were not comprehensively considered, which was identified as a source of personal exposure [28, 29]. It is reliable for children and the elderly, because it is unlikely for them to change the residence frequently in a certain period of time, and they spent less time outdoors compared with adults [30, 31]. However, time spent on commuting or around work place should not be ignored for adults, and exposure on days off work was different from that on workdays. Previous research had investigated daily space–time-activity-weighted model to estimate short-term individual exposure [32], and its association with cardiopulmonary outcomes changed with additional consideration of space–time activity information compared with crude estimation. This method is relatively easy to implement for short-term exposure. But as for long-term exposure, important personal day-to-day activity should also be considered. Previous studies had characterized personal exposure to PM_2.5_ [21, 27], however, individual time-weighted PM_10_ exposure was not fully calculated.

Ambient PM would induce the mortality and morbidity of sudden cardiac and cerebrovascular events by promoting the progression of atherosclerotic plaque and heightened thrombogenicity. This is a long-term progression with interacted and joint adverse effect of multiple factors [25]. Ambient air pollution is a mixture of particles and gases. These pollutants are highly correlated, and their combined effects on health exist. Double or multi-pollutant models were performed to obtain their independent effect [26–31, 33]. But the overall effect of co-exposure to pollutants cannot be interpreted, and high collinearity cannot be avoided. Quantile g-computation was presented to eliminate potential errors arising from collinearity by weighted method and obtain the total effect [34, 35].

In this study, we calculated one- and two-year time-weighted average exposures to PM_2.5_ and PM_10_ based on LUR model considering personal activity pattern (commuting mode, physical activity modes, residential and work addresses, time spent indoors, outdoors and on commuting) and infiltration factors. We aimed to investigate long-term association between time-weighted average exposure to PM_2.5_ and PM_10_ and the risk of carotid atherosclerosis, and further explore the overall effect of co-exposure to ambient pollutants on carotid atherosclerosis.

## Materials and methods

### Participants

**Fig. 1:**
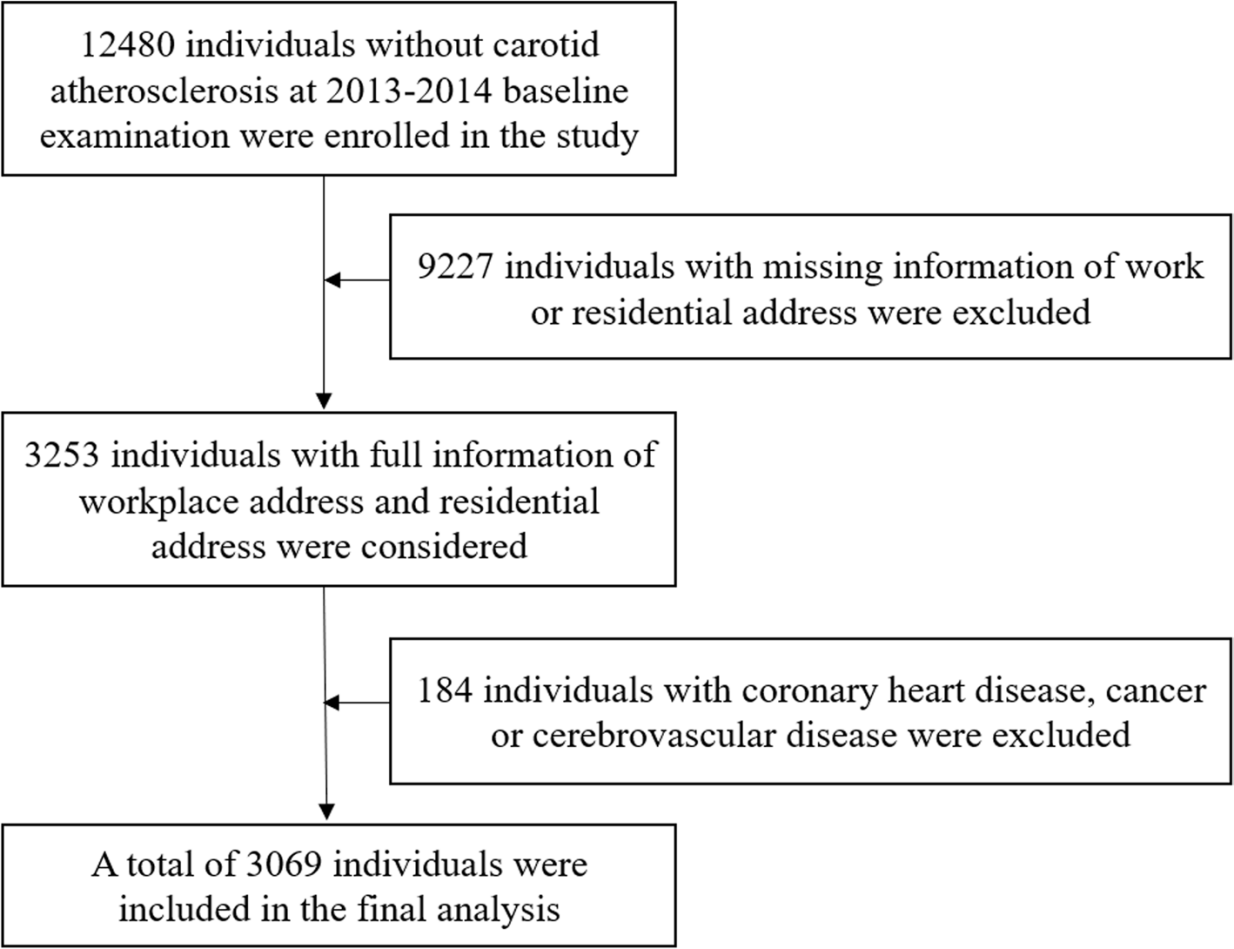
Flow chart about the enrollment of the participants

Study population derived from Beijing Health Management Cohort (BHMC) study. Detailed information of physical examination was recorded each year. The detailed procedure of participant enrollment was described in Fig. 1. Related data was collected from the 2013–2014 baseline survey to December 31^st^, 2020 (*n* = 12,480). To calculate individual exposure to ambient pollutants, only participants with both residential and work addresses were included (*n* = 3253). We further excluded those with coronary heart disease, cancer or cerebrovascular disease (*n* = 184). Finally, a total number of 3069 participants were enrolled.

### Data collection

Anthropometric and laboratory measurements were conducted by trained medical professionals at baseline and for each follow-up visit. This study followed the 1964 Declaration of Helsinki and the later amendments, and was approved by the Ethics Committee of Capital Medical University (NO: 2019SY088). Written informed consent was obtained from all the participants.

Participants were supposed to take off shoes and heavy clothes to take the anthropometric measurements. Body mass index (BMI) was calculated dividing weight (kg) by the square of height (m^2^). Blood pressure (systolic blood pressure (SBP) and diastolic blood pressure (DBP)) was measured in the right arm in the sitting position using electronic sphygmomanometer with at least 5 min’ rest and 30 min’ interval during which time individuals were not allowed to drink any beverages containing caffeine or alcohol. The equipment should be put at the height of heart and the tested arm. Three times’ measurements were performed, and the records were averaged and applied. Mean arterial pressure (MAP) was calculated by two thirds of DBP plus one third of SBP.

Information of demographic, lifestyle, disease and medication history, including age, sex, physical activity intensity (low, moderate and high intensity), education level (lower or equal to high-school degree, college or university degree, and over or equal to postgraduate degree), excessive salt intake (> 6 g/day or ≤ 6 g/day), drinking status (current alcohol drinker), smoking status (current smoker), disease and medication history of hypertension, diabetes and dyslipidemia was collected based on questionnaire. Physical activity intensity is a question item by asking directly “What about your physical activity intensity” containing three options: ① low intensity: no exercise or mild activities, such as walking, dancing or doing Tai Chi, et al.; ② moderate intensity: including jogging, bicycling or climbing, et al.; ③ high intensity: including swimming, skipping rope or racket and balling sports, et al. Smoking status was characterized as never or former, and current smoker, and drinking status as never or former, and current drinker.

Variables calculating time-weighted average concentration including residential and work addresses were also collected based on questionnaires (Additional file A1). The amount of time spent indoors around the residential and work places commonly were recorded directly (hours) within one day. Commuting modes were categorized as ① driving a car or taking a taxi; ② walking, cycling, taking electric car or motorcycle; ③ taking a bus; ④ taking the subway. And time spent on commuting for a single travel was collected directly (hours) or based on the following options which were defined as ① less than 30 min; ② 30–60 min; ③ longer than 60 min. The specific values we applied were 30, 45 and 60 min for each option, respectively. Information about physical activity frequency was collected based on the following question: How many times did you do exercises for a week? The answers included ① one to two times per week; ② three to five times per week; ③ more than five times per week. The values of two, four and six times were applied for this question, respectively, when calculating time-weighted average exposure. The length for a single time of exercise was collected as “How long about your exercise length for a single time of exercise?”. The answers included three items: ① less than 30 min; ② 30 to 60 min; ③ more than 60 min. And 30, 45 and 60 min were finally applied separately. Activity at indoor or outdoor environment was decided based on the type of exercise. For instance, indoor activity includes yoga, swimming and balling sports in gym, et al. And outdoor activity includes jogging, climbing and bicycling, et al.

After at least 12 h’ overnight fasting, blood samples were collected and analyzed. The following indicators, including fasting blood glucose (FBG), high-density lipoprotein cholesterol (HDL-C), low-density lipoprotein cholesterol (LDL-C), triglycerides (TG) and uric acid (UA), were measured by enzymatic method using a chemistry analyzer (Beckman LX 20, Beckman, Brea, CA, America) at the central laboratory.

### Individual time-weighted exposure to PM

Individual exposure to ambient PM was estimated based on a satellite-based LUR model, and the model performance was shown in Table A1. Additionally considering micro-environment exposure sources and exposure time, individual time-weighted average exposure was calculated.

#### Prediction model

LUR model combined machine learning algorithms was performed to predict daily concentrations of ambient pollutants. Explanatory variables included aerosol optical depth (AOD), meteorological variables, land-use, normalized difference vegetation index (NDVI), traffic road, population density and elevation data.

Pollutant concentrations including PM_2.5_, PM_10_ and gaseous pollutants (sulfur dioxide (SO_2_), nitrogen dioxide (NO_2_), carbon monoxide (CO) and ozone (O_3_)) were obtained from Beijing National Environmental Monitoring Center (http://www.bjnemc.cn/) at 35 monitoring stations from 2013 to 2020. The recorded measurements were checked to remove negative values. Valid measurements were aggregated to obtain daily mean values during the study period at each monitoring station. Original AOD data (MCD19A2) were downloaded from the National Aeronautics and Space Administration (NASA) Moderate Resolution Imaging Spectroradiometer (MODIS) (https://ladsweb.modaps.eosdis.nasa.gov/) onboard the Terra and Aqua satellites. AOD values at 550 nm were applied. Based on the new algorithm of multi-angle implementation of atmospheric correction (MAIAC), AODs with similar accuracy for dark and vegetated areas but higher accuracy for brighter areas [36] were obtained. The product could enlarge the number of valid daily AOD values [37]. AODs with the highest quality were recommended. MOD04 and MYD04 products were applied to fill the missing values. Four main meteorological variables (daily mean temperature, two-minute-average wind speed, barometric pressure and relative humidity at 1,000 hPa) were collected from China Meteorological Administration (http://data.cma.cn). Universal kriging method with an appropriate 1-km resolution was applied to estimate missing values. Annual land-use data at a spatial resolution of 1-km in 2015, 2018 and 2020 were obtained from Resource and Environment Science and Data Center (https://www.resdc.cn/). Road traffic data were obtained through OpenStreetMap (http://download.geofabrik.de/). The total cover of each land use type (including water, forest and urban cover) and total road length in buffers within the given radius were calculated, respectively. The radiuses of buffer zones for land-use type were set as 1 km, 2 km, 3 km, 4 km and 5 km, and for road length were 100 m, 200 m, 300 m, 400 m, 500 m, 600 m, 700 m, 800 m, 900 m, 1 km, 2 km and 3 km. The 16-day NDVI data with 1 km × 1 km grid resolution (MOD13A2) was obtained from NASA official website (https://ladsweb.modaps.eosdis.nasa.gov/). ASTER GDEM Version 3 elevation data (a spatial resolution of approximate 30 m’ horizontal posting at the equator) was downloaded from NASA website (https://lpdaac.usgs.gov/products/astgtmv003/). Population density data was collected via Beijing Statistical Yearbook (http://nj.tjj.beijing.gov.cn/nj/).

Residential and work addresses were geocoded based on Maplocation (https://maplocation.sjfkai.com/) and transformed into standard coordinate system. Explanatory variables and dependent variable (pollutant concentration) were matched according to geographical position and time (day or year). Univariate regression was performed to primarily select explanatory variables linking to pollutant concentration (*P* value for regression coefficient < 0.05). Considering the nonlinear relationship between explanatory variables and pollutant concentration, generalized additive model (GAM) was applied to select variables with better explanatory ability. Predictors were individually added to the model, and the inclusion should not turn over the predefined direction of both individual explanatory variable and the former model. Variance inflation factor (VIF) > 4 was treated as collinearity [38]. We fitted extreme gradient boosting (XGBoost) model with tenfold cross-validation, and the prediction model yielded a mean R^2^ of 0.787 for PM_2.5_ and 0.715 for PM_10_ (Table A1).

#### Calculation of time-weighted individual concentration

We calculated one- and two-year time-weighted average exposure to ambient pollutants based on the following equations [39].

1 $${Y}_{air}=\left({C}_{hout}\ast{T}_{hout}+{C}_{wout}\ast{T}_{wout}+{IF}_{h}\ast\left({C}_{hout}\ast{T}_{hin}+{C}_{wout}\ast{T}_{win}\right)+{T}_{trans}\ast{IF}_{t}\ast\left({C}_{hout}+{C}_{wout}\right)/2\right)/24$$

2 $${Y}_{air}=\left({C}_{hout}\ast{T}_{hout}+{IF}_{h}\ast{C}_{hout}\ast{T}_{hin}+{T}_{trans}\ast{IF}_{t}\ast{C}_{hout}\right)/24$$

Specifically, Eq. (1) illustrated the method of time-weighted pollutant estimation on working days, and Eq. (2) on days off work. In the above-mentioned equations, time scale is deemed as days. In detail, ${C}_{hout}$ and ${C}_{wout}$ represent outdoor concentrations estimated based on LUR model according to individual longitudes and latitudes of geocoded residential and work addresses, respectively; ${T}_{hout}$ and ${T}_{hin}$ represent time spent outside and inside around the residences, respectively; ${T}_{wout}$ and ${T}_{win}$ represent time spent outside and inside around work places, respectively; ${T}_{trans}$ represents the time spent on commuting; ${IF}_{h}$ and ${IF}_{t}$ represent infiltration factors for ambient air pollutants infiltrating into indoors and different traffic vehicles, respectively [40–42]. Exposure on different traffic vehicles was estimated by the average level of infiltrated ambient pollutant around outside residential and outside work places $[\left({C}_{hout}+{C}_{wout}\right)/2]$. Missing values of time spent on commuting were filled in based on the exposure handbook among Chinese population [43] and multiple imputation method. Multivariate imputation via chained equation was performed and five imputed datasets were generated based on an imputing regression model to fill in missing values. Because we did not find suitable infiltration factor of bus for PM_10_, the infiltration factor of bus for PM_2.5_ was applied, instead. Results of correlation analysis between individual pollutant exposure estimated based on time-weighted method, residential and work address were shown in Table A2 (Additional file), and the significant association was found between PM_2.5_ and PM_10_ estimation of time-weighted method and at residential address.

For those diagnosed as carotid atherosclerosis during the follow-up, one- (365-day) and two-year (730-day) time-weighted average exposure to PM_2.5_ and PM_10_ was calculated before the date of diagnosis for carotid atherosclerosis. Similarly, three-, four- and five-year average exposure were also obtained. Individuals without carotid atherosclerotic lesion during the follow-up were treated as censoring, and time-weighted concentration was calculated before the date of the last follow-up.

### Definition of carotid arteriosclerosis

Carotid artery ultrasonography measurement was performed for the diagnosis of carotid atherosclerosis by trained physicians using a 3.5-MHz transducer (logic Q700 MR, GE, Milwaukee, WI, USA) at baseline and during follow-up. Participants were required to be in a supine position. Shortly, bilateral examination on both right and left carotid arteries was carried out using a standardized approach at common carotid artery, external carotid artery and internal carotid artery. Carotid bifurcation and bulb were also measured. CIMT was determined by measuring the distance between the echo fronts of the media adventitia and lumen intima at four different locations (right, left, near walls and far walls). Carotid plaque was characterized as focal thickening of IMT greater than 1.5 mm or a protrusion greater than 50% in relation to neighboring segments [44]. Carotid atherosclerosis was defined by the presence of carotid plaque or focal thickening of the carotid artery wall [45]. Detailed procedure and criteria were rigorously performed according to Mannheim consensus [46].

### Statistical analysis

Baseline characteristics were presented as number (percentage) for categorical variables and mean (standard deviation (SD)) for normal distributed continuous variables or median (interquartile range (IQR)) for continuous variables that did not follow normal distribution. Both of the two pollutants (PM_2.5_ and PM_10_) were categorized into 4 groups according to the quartiles. Stepwise Cox regression models were performed to explore the association between the risk of carotid atherosclerosis and individual exposure to PM_2.5_ and PM_10_ in both continuous and categorical form. Hazard ratios (HRs) and 95% confidence intervals (CIs) were presented for per IQR increase in pollutant concentrations. Model 1 was adjusted for age and sex. Model 2 further controlled education level, smoking status, drinking status, physical activity intensity, excessive salt intake and medication history. Model 3 was additionally adjusted for BMI, MAP, FBG, UA, LDL-C, HDL-C and TG based on model 2. Since the exposure–response trend could be evaluated by modelling PM as a linear term, spline was set for violations of linearity assumption to accommodate potential non-linear relationship. Multivariable adjusted restricted cubic spline model was conducted to assess the exposure–response relationship between time-weighted average exposure to PM_2.5_ and PM_10_ and the risk of carotid atherosclerosis. The number of knots between 3 and 7 were selected and finally decided by comparing Akaike information criterion. Figure results were derived from restricted cubic spline model with 3 degrees of freedom.

Quantile g-computation was performed to evaluate the total effect of multiple ambient pollutant exposures on carotid atherosclerosis. Quantile g-computation is a parametric, generalized-linear model-based method for estimating mixing effect that makes use of g computation. It also supports non-linear and non-additive effects of individual and mixture pollutants, as well as exposures that are associated with study outcome in different directions or with positive or negative weights. Coefficients > 0 for the individual pollutant indicates positive weight; coefficients < 0 indicates negative weight. The overall estimation is the sum of coefficients for pollutants corresponding to the change in risk of carotid atherosclerosis. In this work, quantile g-computation is used to describe the overall risk of carotid atherosclerosis controlling for covariates and give separate importance of each pollutant (PM_2.5_, PM_10_, SO_2_, NO_2_, CO and O_3_). The estimated overall coefficients of all exposures increasing by one quartile at the same time and weights that indicate the contribution of each individual component to the overall estimate were obtained.

We performed several sensitivity analyses described as follows: ① Individuals with only work address were included in model 3 (*n* = 11840); ② Individuals without medication history of hypertension, diabetes and hyperlipidemia were included in model 3 (*n* = 2606); ③ Individual time-weighted average exposure to ambient pollutants was estimated without consideration of infiltration factors; ④ We further adjusted for other pollutant and ran two-pollutant models according to the results of correlation analysis (Significant correlation was deemed as correlation coefficient larger than 0.6 or smaller than -0.6, and *P* value < 0.05) (Table A3). ⑤ Individuals with complete imputed information of calculating time-weighted concentrations were included for the analysis (*n* = 836). We also investigated associations between PM_2.5_ and PM_10_ and risk of carotid atherosclerosis for subgroups defined by age (≥ 60 years or < 60 years), sex (male or female), LDL-C (≥ 3.4 mmol/L or < 3.4 mmol/L), TG (≥ 1.7 mmol/L or < 1.7 mmol/L), HDL-C (≥ 1.0 mmol/L or < 1.0 mmol/L), BMI (≥ 24 kg/m^2^ or < 24 kg/m^2^) and hyperuricemia (yes or no). Subgroup factors for dyslipidemia were collected based on guidelines among Chinese [47]. Hyperuricemia was defined as ≥ 360 μmol/L in women and ≥ 420 μmol/L in men.

For all analyses, a two-tailed *P* value < 0.05 was considered to be statistically significant. Stepwise Cox regression analysis was performed by SAS version 9.4 (SAS Institute, Cary, North Carolina, USA), and multivariable adjusted restricted cubic spline model and quantile g-computation by R version 4.1.1.

## Results

A total of 3069 individuals were included, and 981 (31.96%) individuals diagnosed as carotid atherosclerosis during the study were observed (Table 1). At baseline, the median (IQR) age of the participants was 42 (34–49) years. Nearly half of the participants were men (46.27%). Most of the participants got the college or university degree (76.34%) and had a relatively low physical activity intensity (64.03%). Most participants were nonsmokers (81.13%), nondrinkers (62.63%), and had a relatively lower salt intake (72.94%). A total of 463 individuals had a medication history of hyperlipidemia (*n* = 117), hypertension (*n* = 366) or diabetes (*n* = 117). The median concentration of one- and two-year time-weighted average exposure to PM_2.5_ was 41.899 µg/m^3^ and 41.997 µg/m^3^, and 32.526 µg/m^3^ and 32.495 µg/m^3^ for PM_10_ exposure, respectively (Table 2). Concentration of time-weighted average exposure to PM_10_ is lower than PM_2.5_ due to the application of infiltration factors. Air pollutant exposures within the same time window were positively correlated, except for O_3_, which was negatively or positively correlated with PM_2.5_ and PM_10_ (Table A2).

**Table 1:** Characteristics of the study participants

Variables	Total participants
No. of participants, n	3069
Carotid Atherosclerosis, n (%)	981 (31.96)
Male, n (%)	1420 (46.27)
Age (years), median (IQR)	42 (34–49)
Age ≥ 60, n (%)	197 (6.42)
Education level, n (%)	
Lower or equal to high-school degree	216 (7.04)
College or university degree	2343 (76.34)
Over or equal to postgraduate degree	510 (16.62)
Physical activity intensity, n (%)	
Low	1965 (64.03)
Moderate	923 (30.07)
High	181 (5.90)
Current smoker, n (%)	579 (18.87)
Current alcohol drinker, n (%)	1147 (37.37)
Excessive salt intake (> 6 g/day), n (%)	830 (27.04)
Medication history of dyslipidemia, n (%)	117 (3.81)
Medication history of hypertension, n (%)	366 (11.93)
Medication history of diabetes, n (%)	117 (3.81)
FBG (*mmol/L*), median (IQR)	5.18 (4.88–5.55)
MAP (mmHg), median (IQR)	90.00 (81.67–96.67)
BMI (kg/m^2^), median (IQR)	24.16 (21.87–26.42)
LDL-C (mmol/L), median (IQR)	2.74 (2.28–3.22)
TG (mmol/L), median (IQR)	1.16 (0.78–1.82)
HDL-C (mmol/L), median (IQR)	1.34 (1.12–1.58)
UA (μmol/L), median (IQR)	288.71 (232.00–357.00)

*IQR* interquartile range, *FBG* fasting blood glucose, *MAP* mean arterial pressure, *BMI* body mass index, *LDL-C* low-density lipoprotein cholesterol, *TG* triglycerides, *HDL-C* high-density lipoprotein cholesterol, *UA* uric acid, *PM*_*2.5*_ particulate matter with aerodynamic diameter < 2.5 μm, *PM*_*10*_ particulate matter with aerodynamic diameter < 10 μm

**Table 2:** Descriptive statistics of individual time-weighted exposure to ambient PM

Ambient PM	Mean (SD)	Min	Max	Percentiles				
				5th	25th	50th	75th	95th
PM_2.5_ (μg/m^3^)								
1-year exposure	42.276 (3.843)	31.435	74.004	37.080	39.669	41.899	44.494	48.426
2-year exposure	42.414 (3.940)	31.809	74.243	36.902	39.703	41.997	44.644	48.898
PM_10_ (μg/m^3^)								
1-year exposure	32.321 (4.075)	20.819	53.331	24.910	30.091	32.526	34.850	38.807
2-year exposure	32.373 (4.043)	21.146	53.343	25.088	30.172	32.495	34.902	38.685

*SD* standard deviation, *PM* particulate matter, *PM*_*2.5*_ particulate matter with aerodynamic diameter < 2.5 μm, *PM*_*10*_ particulate matter with aerodynamic diameter < 10 μm

**Fig. 2:**
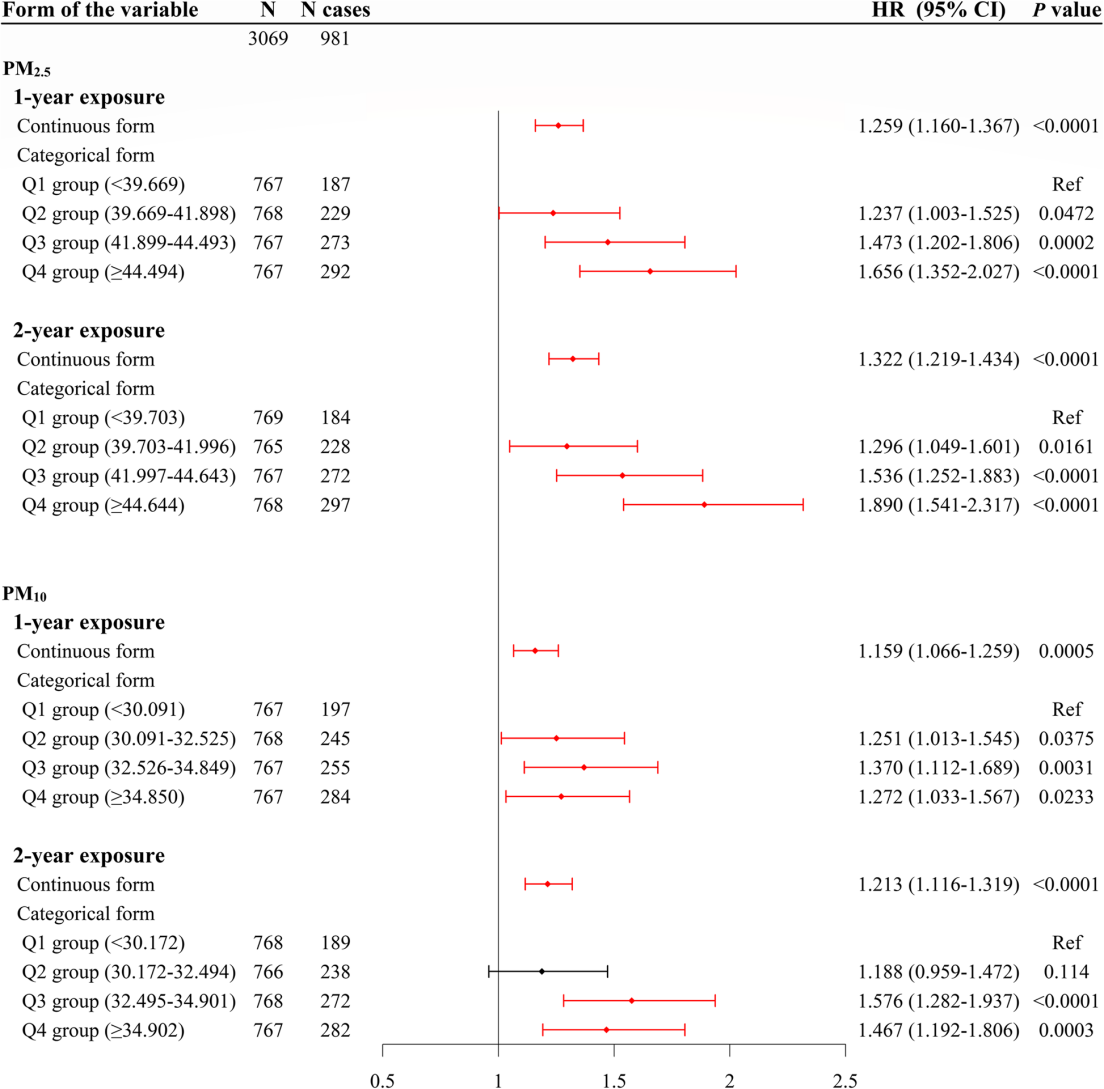
Relationship between individual time-weighted average exposure to PM_2.5_ and PM_10_ and carotid atherosclerosis. PM_2.5_, particulate matter with aerodynamic diameter < 2.5 μm; PM_10_, particulate matter with aerodynamic diameter < 10 μm; Q, quartile; HR, hazard ratio; CI, confidence interval; Ref, reference

The association between time-weighted average exposure to PM_2.5_ and PM_10_ and the risk of carotid atherosclerosis was significant (Table A4-A5, Fig. 2). Time-weighted individual one-year average exposure to PM_2.5_ and PM_10_ was associated with 28.1% and 16.9% increased risk of carotid atherosclerosis based on the crude model. Similar results were obtained for two-year exposure (HR (95% CI) for PM_2.5_ and PM_10_ were 1.335 (1.246–1.429) and 1.219 (1.130–1.314), respectively). The relatively smaller effect sizes were obtained for extended models adjusting for important covariates. The highest quartile (versus the lowest quartile) of PM_2.5_ was associated with the highest risk of carotid atherosclerosis. Effect sizes [HR (95% CI)] for increasing quartiles of time-weighted one-year average exposure to PM_2.5_ were 1.237 (1.003–1.525), 1.473 (1.202–1.806) and 1.656 (1.352–2.027), and of two-year exposure were 1.296 (1.049–1.601), 1.536 (1.252–1.883) and 1.890 (1.541–2.317) of the three higher quartile groups, respectively. The association of PM_10_ with carotid atherosclerosis were also presented when modeling PM_10_ as categorical variable for one- and two-year exposure [HR (95% CI): 1.251 (1.013–1.545), 1.370 (1.112–1.689) and 1.272 (1.033–1.567) for one-year exposure, and 1.188 (0.959–1.472), 1.576 (1.282–1.937) and 1.467 (1.192–1.806) for two-year exposure of the three higher quartile groups, respectively]. Clearly, the association between ambient PM and carotid atherosclerosis existed with further adjustment for personal behavior characteristics and biochemical indicators.

**Fig. 3:**
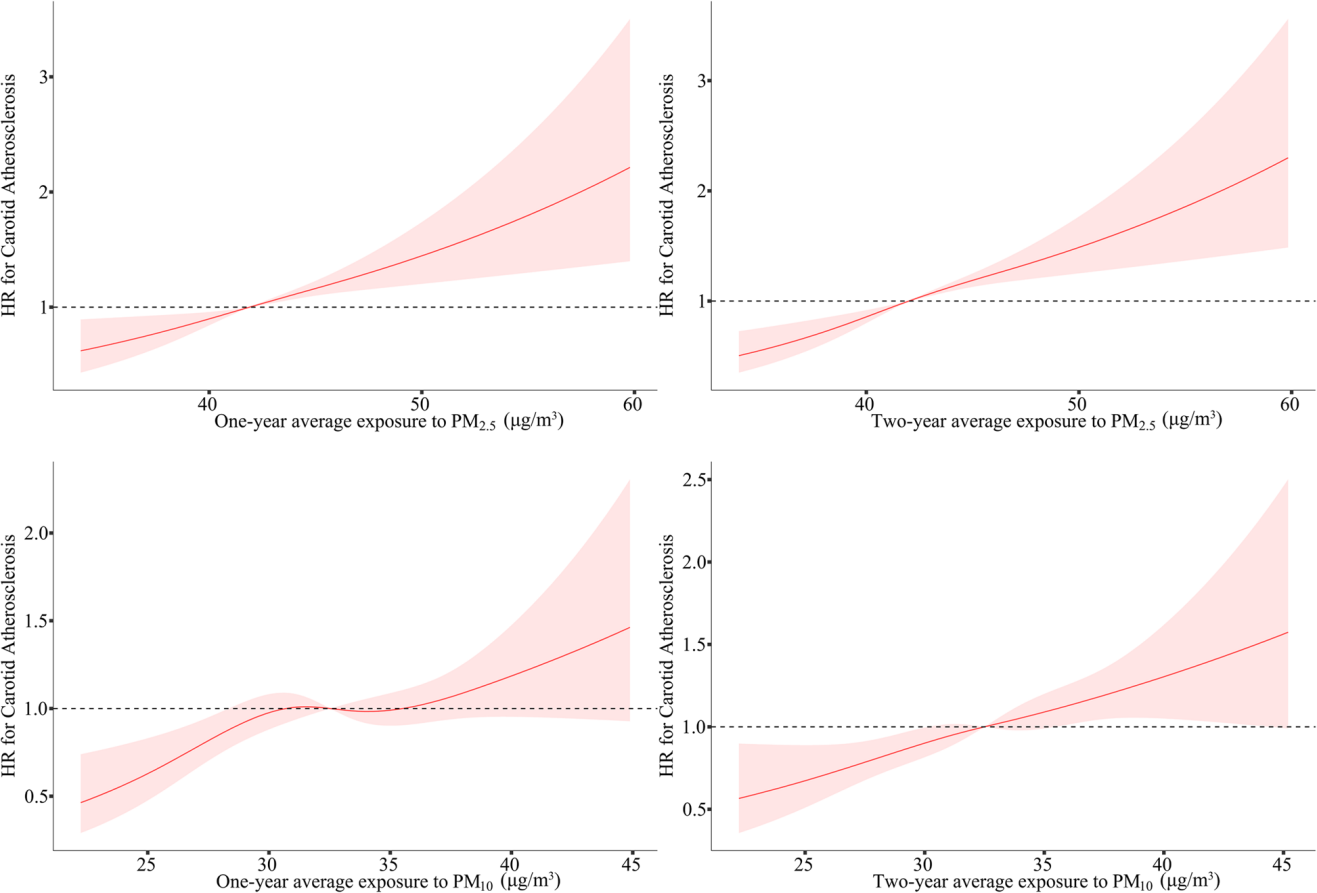
Exposure–response curves for the relationship between individual time-weighted average exposure to PM_2.5_ and PM_10_ and carotid atherosclerosis. **A** and **B** demonstrated the trend for the risk of carotid atherosclerosis with the increasing level of one- and two-year average exposure to PM_2.5_, respectively. **C** and **D** described the exposure–response relationship between one- and two-year average exposure to PM_10_ and the risk of carotid atherosclerosis. PM_2.5_, particulate matter with aerodynamic diameter < 2.5 μm; PM_10_, particulate matter with aerodynamic diameter < 10 μm; HR, hazard ratio

Multivariable Cox regression models with restricted cubic splines showed that the relationship between time-weighted average exposure to PM_2.5_ and the risk of carotid atherosclerosis appeared to be approximately linear across the exposure concentration (Fig. 3). The slope of curves did not show a level off trend at higher level of concentrations in the estimated range. The positive linear relationship was observed of two-year average exposure to ambient PM_10_ over the range of exposure (Fig. 3). The overall upward trend of the risk of carotid atherosclerosis was also shown with increasing PM_10_ concentration (Fig. 3).

Individual time-weighted average exposure to each pollutant contributed to either positive or negative partial effects (Fig. A1) estimated by quantile g-computation approach. The results showed the strong positive weight coefficients of SO_2_, PM_2.5_ and CO. The total positive association between co-exposure to mixed ambient pollutants (PM_2.5_, PM_10_, SO_2_, NO_2_, CO and O_3_) and the risk of carotid atherosclerosis were demonstrated. The risk of carotid atherosclerosis increased by 51% with a change in mixture of time-weighted one-year average exposure to ambient air pollutants by one quartile [HR (95% CI): 1.510 (1.338–1.704)]. The overall mixture effects were driven by SO_2_ (66.7%), followed by CO (13.8%), PM_2.5_ (13.4%), and O_3_ (6.1%). The risk of carotid atherosclerosis was also strongly associated with two-year time-weighted co-exposure to ambient air pollution [HR (95% CI): 1.613 (1.428–1.822)], and the importance of the pollutants was similar to that of one-year exposure [SO_2_ (65.0%), followed by CO (14.7%), PM_2.5_ (13.7%), and O_3_ (6.6%), respectively]. For PM_10_ a strong negative weight was obtained. Detail results of quantile g-computation are shown in Table A6.

Results of sensitivity analysis revealed that our results were robust. The significant relationship between PM_2.5_ and PM_10_ exposure and the risk of carotid atherosclerosis was additionally shown (Table A7-A8). Different exposure period did not change the significant association between PM_2.5_ and PM_10_, except for five-year average exposure. Excluding individuals with missing values of time-weighted method, the results were robust when modeling pollutant as continuous variable. The association between PM_2.5_ exposure and carotid atherosclerosis were independent of NO_2_, O_3_ and CO. However, the significant association between PM_10_ and carotid atherosclerosis disappeared additionally adjusting for CO.

We did not find significant associations between the risk of carotid atherosclerosis and time-weighted average exposure to PM_2.5_ and PM_10_ among subjects who were 60 years or older. We observed the stronger associations between PM_2.5_ and carotid atherosclerosis among males and participants with hyperuricemia, normal level of HDL-C and TG and abnormal level of LDL-C and BMI. Subgroups, which included females and participants with hyperuricemia, normal level of HDL-C and abnormal level of LDL-C, TG and BMI, were more sensitive to PM_10_ exposure with a stronger association with carotid atherosclerosis (Table A9-A10).

## Discussion

We calculated one- and two-year individual time-weighted average exposure to PM_2.5_ and PM_10_ based pollutant estimation from LUR model and a time-weighted method. PM_2.5_ and PM_10_ were identified as risk factors for carotid atherosclerosis. About 32.2% and 21.3% increased risk of carotid atherosclerosis were found with two-year exposure to PM_2.5_ and PM_10_, respectively. The consistent results were obtained for one-year exposure and treating PM as categorical variables. The linear relationship between PM_2.5_ exposure and the risk of carotid atherosclerosis was observed. Such relationship was also shown for PM_10_ exposure. Robust results were demonstrated by a variety of sensitivity analyses. The overall adverse effect of time-weighted co-exposure to ambient pollutants on the risk of carotid atherosclerosis was obtained. Our results suggest that higher levels of PM_2.5_ and PM_10_ exposure would increase the risk of carotid atherosclerosis.

The detailed mechanisms of PM-induced atherosclerotic lesion were not clearly and fully illustrated, but several processes would potentially describe the progression according to mechanistic researches and experiments. Exposure to PM_2.5_ and PM_10_ would promote the release of inflammatory cytokines and reactive oxygen species, induce peroxidative stress and inflammatory process, and further cause endothelial malfunction and vascular remodeling [48, 49]. Tonne et al. had investigated the association between oxidative potential of PM_10_ and CIMT, but it was not statistically significant [50]. More original studies should be performed to investigate the relationship between oxidation and inflammation derived from PM and carotid atherosclerosis. We observed larger effect sizes for PM_2.5_ than PM_10_. It can be explained by greater pulmonary deposition and differences in sources and constituents. Ambient PM_2.5_ primarily emits from fossil combustion, and PM_10_ consists of higher fractions of particles, such as suspended dust. Larger particles had relatively less infiltration efficiency into indoor environment. Our study also demonstrated the significant association between multiple pollutant exposures and the risk of carotid atherosclerosis based on quantile g-computation. Evidence about the association of ambient pollutants with carotid atherosclerosis was enriched.

It was evidenced that increased atherosclerotic burden was observed in areas with higher levels of air pollutants [51]. PM_2.5_ estimated by AOD adjusting for visibility and relative humidity was proved to be associated with atherosclerosis [52]. The significant association of atherosclerosis with interpolated PM_2.5_ was demonstrated among treatment groups of four clinical trials, but not among the whole population or other subgroups [33]. Research derived from Whitehall II study reported the significant association between 52 weeks’ average exposure to PM_10_ estimated by a geostatistical spatiotemporal model with the percent change of CIMT before ultrasound scan [50] without adjustment for important blood biochemical indicators. Study among randomized participants of two rigorous clinical trials also found the significantly higher percent change of CIMT with PM_2.5_ exposure estimated by interpolation model, but some covariates, such as blood lipids, were not controlled [53]. Long-term association between PM_2.5_ and PM_10_ derived from gaussian dispersion model and the prevalence of carotid plaques was significant for an exposed time of lag 15–18 years at three-year follow-up [22]. Among healthy participants of Multicultural Community Health Assessment Trial, the association between PM_2.5_ predicted based on LUR model and the progression of carotid artery atherosclerosis was not significant [26]. A study of midlife population from a prospective cohort found the significant association between estimated individual PM_2.5_ exposure (industry and household emissions) and carotid atherosclerosis, but such relationship was not shown with PM_2.5_ and PM_10_ derived from traffic and heating [23]. Several well-performed cohorts were combined and the overall effect was pooled [54], but the significant associations between PM_2.5_ and PM_10_ and CIMT were not observed.

The results were not totally consistent. Characteristics of the study population is the main source of heterogeneity. BHMC study includes most of individuals taking health examination every year in Beijing. Participants in the same examination group hold the similar socioeconomical characteristics and activity patterns at work. We controlled important metabolic and behavior indicators to explore the independent association of PM with carotid atherosclerosis. Accurate estimation of individual exposure to pollutants would help quantify the actual relationship between PM and carotid atherosclerosis. Compared with previous studies [13, 18, 22], we applied LUR model and a time-weighted method to estimate individual exposure [21, 27]. The association between PM_10_ and PM_2.5_ and cardiopulmonary outcomes became statistically significant when space–time-activity-weighted exposure was calculated. But daily space–time-activity-weighted estimates of PM_10_ was not significantly associated with reduced FVC [32]. Daily activity mode was suspected to confound the relationship of pollution exposures with atherosclerosis, but physical activity as an independent variable measured by metabolic-equivalents-of-task was not a mediator [32]. Physical activity and ambient air pollution have a complex interaction on health condition, and their interacted or combined effect on health should be further assessed. Nonetheless, daily-activity-related information deserves more attention to obtain more accurate estimation of individual exposure to ambient pollution.

Our results demonstrated that women and those with higher level of TG, LDL-C, BMI, UA and HDL-C had higher risk of carotid atherosclerosis when exposing to PM_10_. While men and individuals with lower level of TG were elusively sensitive to PM_2.5_ with higher risk of carotid atherosclerosis. It was once reported that a greater susceptibility for atherosclerotic lesion of ambient PM were interpreted among individuals with dyslipidemia [13], overweight [15] and hyperuricemia. One possible explanation for this finding is the weaker association of PM with carotid atherosclerosis compared with cardiometabolic risk factors. Pre-existing health conditions would also have an effect. Additionally, such results should be explained with caution because of the small sample size. The observed vulnerability of men may be described as more time spent outdoors, larger airways and enhanced deposition of PM.

Our study has several advantages. First, individual exposure to PM was calculated based on LUR model at both residential and work addresses and a time-weighted method, which can better represent personal exposure. Ambient and indoor PM are highly correlated, but infiltrated indoor ambient PM should not be ignored. Second, quantile g-computation was applied to control collinearity of the pollutants, and the overall adverse effects of individual exposure to multiple pollutants were obtained in our study. Our study enriched evidences about the total positive effect of multiple pollutant exposures, which was less explored before.

Some limitations would affect our findings. Household PM is also an important source of individual pollution, such as fuel use and tobacco smoking, but it was not considered in our study due to limited data. Infiltration factors of bus for PM_10_ were not clearly investigated according to current studies. Thus, infiltration factor of bus for PM_2.5_ was applied to approximately represent that of PM_10_, which would induce bias. We collected individual activity information only at the baseline questionnaire. Additionally, small samples in subgroup analysis and enrolling participants only in Beijing would inhibit the result generalization. Due to exclusion of individuals without residential or work address to calculate time-weighted average exposure, the sample size became smaller, which would result in potential selection bias. The negative weight for PM_10_ is difficult to interpret, possibly reflecting co-linearity with SO_2_, which suggests that there is a mixture effect, and the overall effect may be more robust than the weights of individual pollutants. Further researches are required to explore the relationship between real personal exposure to PM and the prevalence and progression of carotid atherosclerosis considering follow-up information among larger sample of population in multicenter studies. The overall effect of co-exposure to ambient air pollutants on carotid atherosclerosis and their respective contribution to overall effect should also be investigated.

## Conclusions

In conclusion, our study provides population-based evidences about long-term association between time-weighted average exposures to PM_2.5_ and PM_10_ and the risk of carotid atherosclerosis. Our findings highlight the role of PM as a risk factor for carotid atherosclerosis proved by the linear or nonlinear exposure–response curves. Besides, the overall adverse effect of multiple pollutants is also proved. These findings contribute to both policy-making on improvement of ambient pollution and prevention of atherosclerosis-related diseases.

## Supplementary Information

**Additional file 1:** Additional file A1 illustrated information for several parts of questionnaire for BHMC study. **Table A1.** Information about predictive model accuracy for each pollutant. PM_2.5_, particulate matter with aerodynamic diameter <2.5 μm; PM_10_, particulate matter with aerodynamic diameter <10 μm; NO_2_, nitrogen dioxide; SO_2_, sulfur dioxide; O_3_, ozone; CO, carbon monoxide; RMSE, root mean square error; MAE, mean absolute error. **Table A2.** Correlation analysis between individual exposure to PM_2.5_ and PM_10_ estimated based on time-weighed method, residential address and work address * *P* values <0.05 for Spearman correlation coefficients. PM_2.5_, particulate matter with aerodynamic diameter <2.5 μm; PM_10_, particulate matter with aerodynamic diameter <10 μm. **Table A3.** Spearman’s correlation coefficients between individual time-weighted average exposure to ambient pollutants * *P* values <0.05 for correlation coefficients. PM_2.5_, particulate matter with aerodynamic diameter <2.5 μm; PM_10_, particulate matter with aerodynamic diameter <10 μm; NO_2_, nitrogen dioxide; SO_2_, sulfur dioxide; O_3_, ozone; CO, carbon monoxide. **Table A4.** Estimated risk for carotid atherosclerosis associated with individual time-weighted average exposure to PM_2.5_^a^ Model 1: adjusted for age and gender.  ^b^ Model 2: adjusted for variables in model 1, as well as education level, smoking status, drinking status, physical activity intensity, excessive salt intake and medication history of hypertension, diabetes and hyperlipidemia.  ^c^ Model 3: adjusted for variables in model 2 plus BMI, MAP, UA, FBG, LDL-C, HDL-C and TG. Ref, Reference; PM_2.5_, particulate matter with aerodynamic diameter <2.5 μm; Q, quartile; HR, hazard ratio; CI, confidence interval; IQR, interquartile range; FBG, fasting blood glucose; MAP, mean arterial pressure; BMI, body mass index; LDL-C, low-density lipoprotein cholesterol; TG, triglycerides; HDL-C, high-density lipoprotein cholesterol; UA, uric acid. **Table A5.** Estimated risk for carotid atherosclerosis associated with individual time-weighted average exposure to PM_10_^a^ Model 1: adjusted for age and gender. ^b^ Model 2: adjusted for variables in model 1, as well as education level, smoking status, drinking status, physical activity intensity, excessive salt intake and medication history of hypertension, diabetes and hyperlipidemia. ^c^ Model 3: adjusted for variables in model 2 plus BMI, MAP, UA, FBG, LDL-C, HDL-C and TG. Ref, Reference; PM_10_, particulate matter with aerodynamic diameter <10 μm; Q, quartile; HR, hazard ratio; CI, confidence interval; IQR, interquartile range; FBG, fasting blood glucose; MAP, mean arterial pressure; BMI, body mass index; LDL-C, low-density lipoprotein cholesterol; TG, triglycerides; HDL-C, high-density lipoprotein cholesterol; UA, uric acid. **Table A6.** Estimated risk for carotid atherosclerosis in multi-pollutant models according to quantile g-computation. Covariates in model 3 were adjusted for. Coefficient indicates the separate effect among the total effect for individual pollutants. Weight indicates the importance of each pollutant which has the same effect direction. Estimate indicates the combined estimated coefficient. PM_2.5_, particulate matter with aerodynamic diameter <2.5 μm; PM_10_, particulate matter with aerodynamic diameter <10 μm; NO_2_, nitrogen dioxide; SO_2_, sulfur dioxide; O_3_, ozone; CO, carbon monoxide; HR, hazard ratio; CI, confidence interval. **Table A7. **Sensitivity analyses for the association between PM_2.5_ exposure and carotid atherosclerosis Variables was adjusted for in model 3. - Because of high correlation and collinearity, two-pollutant model was not conducted. ^a^ The estimation of individual exposure did not consider pollutant infiltration from outdoor to indoor environment. ^b^ Individuals with complete work address (*n*=11843) were included in the analysis. ^c^ Individuals with missing values of time-weighted variables were excluded (*n*=836). Ref, Reference; PM_2.5_, particulate matter with aerodynamic diameter <2.5 μm; Q, quartile; HR, hazard ratio; CI, confidence interval; NO_2_, nitrogen dioxide; SO_2_, sulfur dioxide; O_3_, ozone; CO, carbon monoxide. **Table A8.** Sensitivity analyses for the association between PM_10_ exposure and carotid atherosclerosis Variables was adjusted for in model 3. - Because of high correlation and collinearity, two-pollutant model was not conducted. ^a^ The estimation of individual exposure did not consider pollutant infiltration from outdoor to indoor environment. ^b^ Individuals with complete work address (*n*=11843) were included in the analysis. ^c^ Individuals with missing values of time-weighted variables were excluded (*n*=836). Ref, Reference; PM_10_, particulate matter with aerodynamic diameter <10 μm; Q, quartile; HR, hazard ratio; CI, confidence interval; NO_2_, nitrogen dioxide; O_3_, ozone; CO, carbon monoxide. **Table A9.** Subgroup analyses exploring the association between individual PM_2.5_ exposure and carotid atherosclerosis Variables was adjusted for in model 3. Ref, Reference; PM_2.5_, particulate matter with aerodynamic diameter <2.5 μm; Q, quartile; HR, hazard ratio; CI, confidence interval; BMI, body mass index; LDL-C, low density lipoprotein cholesterol; TG, triglycerides; HDL-C, high-density lipoprotein cholesterol. **Table A10.** Subgroup analyses exploring the association between individual PM_10_ exposure and carotid atherosclerosis Variables was adjusted for in model 3. Ref, Reference; PM_10_, particulate matter with aerodynamic diameter <10 μm; Q, quartile; HR, hazard ratio; CI, confidence interval; BMI, body mass index; LDL-C, low-density lipoprotein cholesterol; TG, triglycerides; HDL-C, high-density lipoprotein cholesterol. **Fig. A1.** Combined effect of personal time-weighted average exposure to mixed ambient pollutants on the risk of carotid atherosclerosis. A and B represented the weight of one- and two-year co-exposure to ambient air pollutants on the risk of carotid atherosclerosis, respectively, according to quantile g-computation regression. PM_10_, particulate matter with aerodynamic diameter <10 μm; PM_2.5_, particulate matter with aerodynamic diameter <2.5 μm; NO_2_, nitrogen dioxide; SO_2_, sulfur dioxide; O_3_, ozone; CO, carbon monoxide.

## Data Availability

The datasets are not publicly available because of the private information, but could be accessed from the corresponding author with reasonable request.

## Abbreviations

PM: Particulate matter
PM_10_: Particulate matter with aerodynamic diameter < 10 μm
PM_2.5_: Particulate matter with aerodynamic diameter < 2.5 μm
HR: Hazard ratio
CI: Confidence interval
CVDs: Cardiovascular diseases
CIMT: Carotid intima-media thickness
LUR: Land-use regression
MESA Air: Multi-Ethnic Study of Atherosclerosis and Air Pollution
BHMC: Beijing Health Management Cohort
BMI: Body mass index
MAP: Mean arterial pressure
FBG: Fasting blood glucose
HDL-C: High-density lipoprotein cholesterol
LDL-C: Low-density lipoprotein cholesterol
TG: Triglycerides
UA: Uric acid
SO_2_: Sulfur dioxide
NO_2_: Nitrogen dioxide
CO: Carbon monoxide
O_3_: Ozone
SD: Standard deviation
IQR: Interquartile range
