# Supplementary material for: Associations of time-weighted individual exposure to ambient particulate matter with carotid atherosclerosis in Beijing, China
Source: Environ Health. 2023 May 29;22:45. doi: 10.1186/s12940-023-00995-8 (PMC10226216; doi:10.1186/s12940-023-00995-8)
Supplement: Supplementary file 1 — Additional file 1: Additional file A1 illustrated information for several parts of questionnaire for BHMC study. Table A1. Information about predictive model accuracy for each pollutant. PM2.5, particulate matter with aerodynamic diameter <2.5 μm; PM10, particulate matter with aerodynamic diameter <10 μm; NO2, nitrogen dioxide; SO2, sulfur dioxide; O3, ozone; CO, carbon monoxide; RMSE, root mean square error; MAE, mean absolute error. Table A2. Correlation analysis between individual exposure to PM2.5 and PM10 estimated based on time-weighed method, residential address and work address * P values <0.05 for Spearman correlation coefficients. PM2.5, particulate matter with aerodynamic diameter <2.5 μm; PM10, particulate matter with aerodynamic diameter <10 μm. Table A3. Spearman’s correlation coefficients between individual time-weighted average exposure to ambient pollutants * P values <0.05 for correlation coefficients. PM2.5, particulate matter with aerodynamic diameter <2.5 μm; PM10, particulate matter with aerodynamic diameter <10 μm; NO2, nitrogen dioxide; SO2, sulfur dioxide; O3, ozone; CO, carbon monoxide. Table A4. Estimated risk for carotid atherosclerosis associated with individual time-weighted average exposure to PM2.5 a Model 1: adjusted for age and gender. b Model 2: adjusted for variables in model 1, as well as education level, smoking status, drinking status, physical activity intensity, excessive salt intake and medication history of hypertension, diabetes and hyperlipidemia. c Model 3: adjusted for variables in model 2 plus BMI, MAP, UA, FBG, LDL-C, HDL-C and TG. Ref, Reference; PM2.5, particulate matter with aerodynamic diameter <2.5 μm; Q, quartile; HR, hazard ratio; CI, confidence interval; IQR, interquartile range; FBG, fasting blood glucose; MAP, mean arterial pressure; BMI, body mass index; LDL-C, low-density lipoprotein cholesterol; TG, triglycerides; HDL-C, high-density lipoprotein cholesterol; UA, uric acid. Table A5. Estimated risk for car [file 12940_2023_995_MOESM1_ESM.docx]

**Additional file A1**

The questionnaire was consisted of eleven items, including basic information, menstrual and reproductive history, history of current illness, family history of disease, lifestyle habits, air pollution, sleep condition, physical symptom (last three months), mental health, fatigue and sociological scales. The questionnaires were collected by two trained researchers simultaneously for data check to guarantee accuracy and reliability. The original information of questionnaire was collected into several parts. We applied contents of basic information, history of current illness, lifestyle habits and air pollution for this study.

**A1.1 Basic information**

A1.1.1 Sex: 1. Male; 2. Female

A1.1.2 Birth date: <yyyy/mm/dd>

A1.1.3 Occupation: 1. Worker; 2. Farmer; 3. Personnel at official department; 4. Attendant or salesperson; 5. Professional and technical staff; 6. Student; 7. Housekeeper; 8. Retiree; 9. Other_____

A1.1.4 Marriage:1. Unmarried; 2. Married; 3. Divorced; 4. Widowed

A1.1.5 Education level: 1. Lower or equal to high-school degree; 2. College or university degree; 3. Over or equal to postgraduate degree

A1.1.6 Native or not: 1. Yes; 2. No

A1.1.7 Cumulative residence: 1. Less than 3 years; 2. 3-5 years; 3. 6-10 years; 4. 10 years or more

A1.1.8 Your current place of residence: ____(district/county) __ street

A1.1.9 Your current work place: ____(district/county) __ street

**A1.2 History of current illness**

A1.2.1 Suffering from the following diseases or not: 1. Yes; 2. No; 3. Not known

A1.2.1.1 Hypertension: 1. Yes (Maximum blood pressure: _/_mmHg; Date of examination: __; Receiving western medicine or not: 1. Yes; 2. No); 2. No

A1.2.1.2 Diabetes: 1. Yes (Maximum blood glucose: __mmol/L; Date of examination: __; Receiving western medicine or not: 1. Yes; 2. No); 2. No

A1.2.1.3 Coronary heart disease: 1. Yes (Date of diagnosis: __; Receiving western medicine or not: 1. Yes; 2. No); 2. No

A1.2.1.4 Stroke: 1. Yes (Date of diagnosis: __; Receiving western medicine or not: 1. Yes; 2. No); 2. No

A1.2.1.5 Chronic kidney disease: 1. Yes (Date of diagnosis: __; Receiving western medicine or not: 1. Yes; 2. No); 2. No

A1.2.1.6 Chronic obstructive pulmonary disease: 1. Yes (Date of diagnosis: __; Receiving western medicine or not: 1. Yes; 2. No); 2. No

A1.2.1.7 Dyslipidemia: 1. Yes (Date of diagnosis: __; Receiving western medicine or not: 1. Yes; 2. No); 2. No

A1.2.1.8 Asthma: 1. Yes (① Date of diagnosis: __; ② Receiving western medicine or not: 1. Yes; 2. No; ③ Type of asthma diagnosis: 1. Allergic asthma; 2. Cardiogenic asthma; 3. Other: ___); 2. No

A1.2.2 Presence of malignant tumor: 1. Yes; 2. No; 3. not known

A1.2.2.1 Type of malignant tumor:

Lung cancer: 1. Yes; 2. No;

Liver cancer: 1. Yes; 2. No;

Gastric cancer: 1. Yes; 2. No;

Esophageal cancer: 1. Yes; 2. No;

Other: 1. Yes: __; 2. No

A1.2.2.2 Date of diagnosis: ____

A1.2.3 Previous therapy: 1. Untreated; 2. Surgery; 3. Radiotherapy; 4. Chemotherapy; 5. Other therapy:1. Yes; __ 2. No

A1.2.4 Normative medication use:1. Yes; __ 2. No

A1.2.5 Uric acid-lowering drugs:1. Yes: __; 2. No;

Anti-arrhythmic drugs:1. Yes: __; 2. No;

Antipyretics:1. Yes: __; 2. No;

Hormones:1. Yes: __; 2. No;

Diuretics:1. Yes: __; 2. No;

Chinese herbs:1. Yes: __; 2. No;

Sedatives or sleeping pills:1. Yes: __; 2. No;

Antidepressants:1. Yes: __; 2. No;

Other: 1. Yes: ______; 2. No

A1.2.6 Allergy:1. Yes; __ 2. No; 3. Not known

A1.2.7 Penicillin allergy:1. Yes; 2. No;

Sulforaphane allergy:1. Yes; 2. No;

Streptomycin allergy:1. Yes; 2. No;

Cephalosporin allergy:1. Yes; 2. No;

Allergy to egg:1. Yes; 2. No;

Allergy to milk :1. Yes; 2. No;

Allergy to seafood:1. Yes; 2. No;

Allergy to pollen or dust mites:1. Yes; 2. No;

Allergy to detergent:1. Yes; 2. No;

Allergy to dust:1. Yes; 2. No;

Allergy to cosmetic:1. Yes; 2. No;

Other allergies:1. Yes: __; 2. No;

**A1.3 Lifestyle habits**

A1.3.1 Smoke: 1. Never smoking or former smoker (Quitting smoking at age of__ years old; Beginning smoking at__ years old; Smoking __ cigarettes per day before quitting); 2. Current smoker (Starting smoking at __ years old; Smoking __ cigarettes per day)

A1.3.2 Reason for quitting smoking: 1. Active; 2. Passive

A1.3.3 Reason for quitting passive smoking: 1. Pregnancy/preparation for pregnancy; 2. Illness; 3. Avoidance of family conflicts; 4. Reason for working; 5. Other: __

A1.3.4 Passive smoking: 1. Never; 2. As a passive smoker for __ years, stopping passive smoking on <yyyy/mm/dd>; 3. As a current passive smoker for __ years, accumulative passive smoking __ hours per day

A1.3.5 Frequency of passive smoking: 1. <1 day/week; 2. 1-2 days/week; 3. 3-4 days/week; 4. Almost everyday

A1.3.6 Drinking alcohol: 1. Never drinking or former drinker (Quitting drinking for __ years); 2. Current drinker (Starting drinking at __ years old)

A1.3.7 Type of alcohol consumed: 1. White wine; 2. Beer; 3. Red wine; 4. All of the above-mentioned

A1.3.8 Frequency of drinking alcohol per week: 1. 1-2 times; 2. 3-4 times; 3. ≥5 times

A1.3.9 Amount of alcohol for each time: 1. <50 grams; 2. 50-100 grams; 3. 150-200 grams; 4. ≥250 grams

A1.3.10 Excessive salt intake: 1. > 6g/day; 2. ≤ 6g/day

A1.3.11 Physical activity intensity: 1. Low intensity: no exercise or mild activities, such as walking, dancing or doing Tai Chi, et al.; 2. Moderate intensity: including jogging, bicycling or climbing, et al.; 3. High intensity: including swimming, skipping rope or racket and balling sports, et al.

A1.3.12 Regularly physical activity for __ years

A1.3.13 How many times did you do exercises for a week? 1. 1-2 times per week; 2. 3-5 times per week; 3. ≥ 5 times per week.

A1.3.14 How long about your exercise length for a single time of exercise? 1. Less than 30 minutes; 2. 30 to 60 minutes; 3. More than 60 minutes

A1.3.15 Manual labor: 1. Light; 2. Moderate; 3. Heavy

A1.3.16 Days of work per week: 1. <3 days; 2. 3-5 days; 3. >5 days

A1.3.17 Hours of sitting time per day: 1. <2 hours; 2. 2-4 hours; 3. 4-6 hours; 4. >6 hours

A1.3.18 What is your main mode of transport to work most frequently? 1. Driving a car or taking a taxi; 2. Walking, cycling, taking electric car or motorcycle; 3. Taking a bus; 4. taking the subway.

A1.3.19 How often do you spend on commuting (for the single travel)? 1. Less than 30 minutes; 2. 30-60 minutes; 3. Longer than 60 minutes

**A1.4 Air pollution**

6.1 Time spent outdoors around residential place: __ hours/day

6.2 Time spent outdoors around work place: __ hours/day

6.3 Wearing a mask: 1. Yes; 2. No; 3. Occasionally

6.4 Type of mask: 1. Cotton mask; 2. Medical non-woven mask; 3. Activated carbon mask; 4. Dust mask (including 3M mask / N95 mask)

6.5 Use of air purifier: 1. Yes; 2. No; 3. Occasionally

6.6 Physical discomfort: 1. Yes; 2. No

6.7 Type of physical discomfort: 1. Cough; 2. Asthma; 3. Sore throat; 4. Depression; 5. Other: _____

6.8 Cooking fuel: 1. Natural gas; 2. Coal gas; 3. Coal; 4. Electricity; 5. Other: _____

6.8 Time of cooking: __hours

Note: Because authors had signed nondisclosure agreements, the research data could not be accessed directly. Under the permission of researchers from Beijing Health Management Cohort (BHMC) study, we provided more detailed information about the questionnaire.

| Table A1 Information about predictive model accuracy for each pollutant. | | | |
| --- | --- | --- | --- |
| Pollutant | Adjusted R^2^ | RMSE | MAE |
| PM_2.5_ | 0.787 | 29.610 | 18.569 |
| PM_10_ | 0.715 | 43.174 | 27.347 |
| SO_2_ | 0.802 | 8.421 | 4.617 |
| CO | 0.756 | 0.472 | 0.285 |
| NO_2_ | 0.813 | 12.366 | 8.666 |
| O_3_ | 0.830 | 21.772 | 13.009 |
| PM_2.5_, particulate matter with aerodynamic diameter <2.5 μm; PM_10_, particulate matter with aerodynamic diameter <10 μm; NO_2_, nitrogen dioxide; SO_2_, sulfur dioxide; O_3_, ozone; CO, carbon monoxide; RMSE, root mean square error; MAE, mean absolute error. | | | |

| Table A2 Correlation analysis between individual exposure to PM_2.5_ and PM_10_ estimated based on time-weighed method, residential address and work address | | | | | | | | | | | | | |
| --- | --- | --- | --- | --- | --- | --- | --- | --- | --- | --- | --- | --- | --- |
| Continuous variable | | | | | | | Categorical variable | | | | | | |
| 1-year exposure | | | | | | | 1-year exposure | | | | | | |
|  | | PM_2.5 (home)_ | PM_2.5 (work)_ | PM_2.5 (time-weighted)_ | | |  | | PM_2.5 (home)_ | PM_2.5 (work)_ | | PM_2.5 (time-weighted)_ | |
| PM_2.5 (home)_ | | 1.000 |  |  | | | PM_2.5 (home)_ | | 1.000 |  | |  | |
| PM_2.5 (work)_ | | 0.114^*^ | 1.000 |  | | | PM_2.5 (work)_ | | 0.115^*^ | 1.000 | |  | |
| PM_2.5 (time-weighted)_ | | 0.809^*^ | 0.477^*^ | 1.000 | | | PM_2.5 (time-weighted)_ | | 0.757^*^ | 0.433^*^ | | 1.000 | |
|  | PM_10 (home)_ | | PM_10 (work)_ | | PM_10 (time-weighted)_ |  | | PM_10 (home)_ | | | PM_10 (work)_ | | PM_10 (time-weighted)_ |
| PM_10 (home)_ | 1.000 | |  | |  | PM_10 (home)_ | | 1.000 | | |  | |  |
| PM_10 (work)_ | 0.231^*^ | | 1.000 | |  | PM_10 (work)_ | | 0.253^*^ | | | 1.000 | |  |
| PM_10 (time-weighted)_ | 0.660^*^ | | 0.434^*^ | | 1.000 | PM_10 (time-weighted)_ | | 0.620^*^ | | | 0.417^*^ | | 1.000 |
| 2-year exposure | | | | | | 2-year exposure | | | | | | | |
|  | PM_2.5 (home)_ | | PM_2.5 (work)_ | | PM_2.5 (time-weighted)_ |  | | PM_2.5 (home)_ | | | PM_2.5 (work)_ | | PM_2.5 (time-weighted)_ |
| PM_2.5 (home)_ | 1.000 | |  | |  | PM_2.5 (home)_ | | 1.000 | | |  | |  |
| PM_2.5 (work)_ | 0.153^*^ | | 1.000 | |  | PM_2.5 (work)_ | | 0.144^*^ | | | 1.000 | |  |
| PM_2.5 (time-weighted)_ | 0.809^*^ | | 0.510^*^ | | 1.000 | PM_2.5 (time-weighted)_ | | 0.761^*^ | | | 0.452^*^ | | 1.000 |
|  | PM_10 (home)_ | | PM_10 (work)_ | | PM_10 (time-weighted)_ |  | | PM_10 (home)_ | | | PM_10 (work)_ | | PM_10 (time-weighted)_ |
| PM_10 (home)_ | 1.000 | |  | |  | PM_10 (home)_ | | 1.000 | | |  | |  |
| PM_10 (work)_ | 0.198^*^ | | 1.000 | |  | PM_10 (work)_ | | 0.205^*^ | | | 1.000 | |  |
| PM_10 (time-weighted)_ | 0.654^*^ | | 0.418^*^ | | 1.000 | PM_10 (time-weighted)_ | | 0.618^*^ | | | 0.375^*^ | | 1.000 |
| * *P* values <0.05 for Spearman correlation coefficients.  PM_2.5_, particulate matter with aerodynamic diameter <2.5 μm; PM_10_, particulate matter with aerodynamic diameter <10 μm | | | | | | | | | | | | | |

| Table A3 Spearman’s correlation coefficients between individual time-weighted average exposure to ambient pollutants | | | | | | | | | | | | | |
| --- | --- | --- | --- | --- | --- | --- | --- | --- | --- | --- | --- | --- | --- |
| Continuous variable | | | | | | | Categorical variable | | | | | | |
| 1-year exposure | | | | | | | 1-year exposure | | | | | | |
|  | PM_2.5_ | PM_10_ | SO_2_ | CO | NO_2_ | O_3_ |  | PM_2.5_ | PM_10_ | SO_2_ | CO | NO_2_ | O_3_ |
| PM_2.5_ | 1.000 |  |  |  |  |  | PM_2.5_ | 1.000 |  |  |  |  |  |
| PM_10_ | 0.609^*^ | 1.000 |  |  |  |  | PM_10_ | 0.546^*^ | 1.000 |  |  |  |  |
| SO_2_ | 0.547^*^ | 0.659^*^ | 1.000 |  |  |  | SO_2_ | 0.502^*^ | 0.614^*^ | 1.000 |  |  |  |
| CO | 0.250^*^ | 0.493^*^ | 0.660^*^ | 1.000 |  |  | CO | 0.258^*^ | 0.488^*^ | 0.653^*^ | 1.000 |  |  |
| NO_2_ | 0.244^*^ | 0.632^*^ | 0.592^*^ | 0.661^*^ | 1.000 |  | NO_2_ | 0.237^*^ | 0.612^*^ | 0.587^*^ | 0.633^*^ | 1.000 |  |
| O_3_ | 0.052^*^ | -0.006 | -0.040^*^ | -0.328^*^ | -0.214^*^ | 1.000 | O_3_ | 0.057^*^ | -0.030 | -0.060^*^ | -0.296^*^ | -0.205^*^ | 1.000 |
| 2-year exposure | | | | | | | 2-year exposure | | | | | | |
|  | PM_2.5_ | PM_10_ | SO_2_ | CO | NO_2_ | O_3_ |  | PM_2.5_ | PM_10_ | SO_2_ | CO | NO_2_ | O_3_ |
| PM_2.5_ | 1.000 |  |  |  |  |  | PM_2.5_ | 1.000 |  |  |  |  |  |
| PM_10_ | 0.619^*^ | 1.000 |  |  |  |  | PM_10_ | 0.557^*^ | 1.000 |  |  |  |  |
| SO_2_ | 0.553^*^ | 0.656^*^ | 1.000 |  |  |  | SO_2_ | 0.509^*^ | 0.608^*^ | 1.000 |  |  |  |
| CO | 0.253^*^ | 0.483^*^ | 0.677^*^ | 1.000 |  |  | CO | 0.249^*^ | 0.465^*^ | 0.659^*^ | 1.000 |  |  |
| NO_2_ | 0.227^*^ | 0.600^*^ | 0.596^*^ | 0.671^*^ | 1.000 |  | NO_2_ | 0.219^*^ | 0.582^*^ | 0.580^*^ | 0.649^*^ | 1.000 |  |
| O_3_ | 0.088^*^ | -0.011 | 0.001 | -0.309^*^ | -0.226^*^ | 1.000 | O_3_ | 0.096^*^ | -0.021 | -0.028 | -0.284^*^ | -0.224^*^ | 1.000 |
| * *P* values <0.05 for correlation coefficients.  PM_2.5_, particulate matter with aerodynamic diameter <2.5 μm; PM_10_, particulate matter with aerodynamic diameter <10 μm; NO_2_, nitrogen dioxide; SO_2_, sulfur dioxide; O_3_, ozone; CO, carbon monoxide. | | | | | | | | | | | | | |

| Table A4 Estimated risk for carotid atherosclerosis associated with individual time-weighted average exposure to PM_2.5_ | | | | | | |
| --- | --- | --- | --- | --- | --- | --- |
| Variable | Model 1: HR (95% CI: Lower-Upper) | *P* value | Model 2: HR (95% CI: Lower-Upper) | *P* value | Model 3: HR (95% CI: Lower-Upper) | *P* value |
| Continuous form (μg/m^3^) |  |  |  |  |  |  |
| 1-year exposure | 1.281 (1.194-1.375) | <0.0001 | 1.279 (1.190-1.374) | <0.0001 | 1.259 (1.160-1.367) | <0.0001 |
| 2-year exposure | 1.335 (1.246-1.429) | <0.0001 | 1.333 (1.243-1.430) | <0.0001 | 1.322 (1.219-1.434) | <0.0001 |
| Categorical form (μg/m^3^) |  |  |  |  |  |  |
| 1-year exposure |  |  |  |  |  |  |
| Q1 group (<39.669) | Ref |  | Ref |  | Ref |  |
| Q2 group (39.669-41.898) | 1.203 (0.991-1.460) | 0.0623 | 1.180 (0.971-1.434) | 0.0963 | 1.237 (1.003-1.525) | 0.0472 |
| Q3 group (41.899-44.493) | 1.533 (1.271-1.848) | <0.0001 | 1.511 (1.252-1.824) | <0.0001 | 1.473 (1.202-1.806) | 0.0002 |
| Q4 group (≥44.494) | 1.815 (1.508-2.185) | <0.0001 | 1.752 (1.454-2.110) | <0.0001 | 1.656 (1.352-2.027) | <0.0001 |
| 2-year exposure |  |  |  |  |  |  |
| Q1 group (<39.703) | Ref |  | Ref |  | Ref |  |
| Q2 group (39.703-41.996) | 1.236 (1.017-1.502) | 0.0336 | 1.231 (1.012-1.498) | 0.0375 | 1.296 (1.049-1.601) | 0.0161 |
| Q3 group (41.997-44.643) | 1.623 (1.345-1.959) | <0.0001 | 1.612 (1.335-1.948) | <0.0001 | 1.536 (1.252-1.883) | <0.0001 |
| Q4 group (≥44.644) | 2.087 (1.732-2.513) | <0.0001 | 2.004 (1.662-2.415) | <0.0001 | 1.890 (1.541-2.317) | <0.0001 |
| ^a^ Model 1: adjusted for age and sex.  ^b^ Model 2: adjusted for variables in model 1, as well as education level, smoking status, drinking status, physical activity intensity, excessive salt intake and medication history of hypertension, diabetes and hyperlipidemia.  ^c^ Model 3: adjusted for variables in model 2 plus BMI, MAP, UA, FBG, LDL-C, HDL-C and TG.  Ref, Reference; PM_2.5_, particulate matter with aerodynamic diameter <2.5 μm; Q, quartile; HR, hazard ratio; CI, confidence interval; IQR, interquartile range; FBG, fasting blood glucose; MAP, mean arterial pressure; BMI, body mass index; LDL-C, low-density lipoprotein cholesterol; TG, triglycerides; HDL-C, high-density lipoprotein cholesterol; UA, uric acid. | | | | | | |

| Table A5 Estimated risk for carotid atherosclerosis associated with individual time-weighted average exposure to PM_10_ | | | | | | |
| --- | --- | --- | --- | --- | --- | --- |
| Variable | Model 1^a^: HR (95% CI: Lower-Upper) | *P* value | Model 2^b^: HR (95% CI: Lower-Upper) | *P* value | Model 3^c^: HR (95% CI: Lower-Upper) | *P* value |
| Continuous form (μg/m^3^) |  |  |  |  |  |  |
| 1-year exposure | 1.169 (1.085-1.260) | <0.0001 | 1.157 (1.073-1.248) | 0.0001 | 1.159 (1.066-1.259) | 0.0005 |
| 2-year exposure | 1.219 (1.130-1.314) | <0.0001 | 1.205 (1.117-1.299) | <0.0001 | 1.213 (1.116-1.319) | <0.0001 |
| Categorical form (μg/m^3^) |  |  |  |  |  |  |
| 1-year exposure |  |  |  |  |  |  |
| Q1 group (<30.091) | Ref |  | Ref |  | Ref |  |
| Q2 group (30.091-32.525) | 1.256 (1.039-1.518) | 0.0187 | 1.252 (1.034-1.516) | 0.0213 | 1.251 (1.013-1.545) | 0.0375 |
| Q3 group (32.526-34.849) | 1.289 (1.068-1.557) | 0.0083 | 1.280 (1.058-1.549) | 0.0109 | 1.370 (1.112-1.689) | 0.0031 |
| Q4 group (≥34.850) | 1.333 (1.107-1.604) | 0.0024 | 1.302 (1.080-1.570) | 0.0057 | 1.272 (1.033-1.567) | 0.0233 |
| 2-year exposure |  |  |  |  |  |  |
| Q1 group (<30.172) | Ref |  | Ref |  | Ref |  |
| Q2 group (30.172-32.494) | 1.217 (1.003-1.477) | 0.0471 | 1.246 (1.024-1.516) | 0.0279 | 1.188 (0.959-1.472) | 0.1140 |
| Q3 group (32.495-34.901) | 1.510 (1.251-1.822) | <0.0001 | 1.546 (1.278-1.870) | <0.0001 | 1.576 (1.282-1.937) | <0.0001 |
| Q4 group (≥34.902) | 1.530 (1.268-1.846) | <0.0001 | 1.528 (1.265-1.847) | <0.0001 | 1.467 (1.192-1.806) | 0.0003 |
| ^a^ Model 1: adjusted for age and sex.  ^b^ Model 2: adjusted for variables in model 1, as well as education level, smoking status, drinking status, physical activity intensity, excessive salt intake and medication history of hypertension, diabetes and hyperlipidemia.  ^c^ Model 3: adjusted for variables in model 2 plus BMI, MAP, UA, FBG, LDL-C, HDL-C and TG.  Ref, Reference; PM_10_, particulate matter with aerodynamic diameter <10 μm; Q, quartile; HR, hazard ratio; CI, confidence interval; IQR, interquartile range; FBG, fasting blood glucose; MAP, mean arterial pressure; BMI, body mass index; LDL-C, low-density lipoprotein cholesterol; TG, triglycerides; HDL-C, high-density lipoprotein cholesterol; UA, uric acid. | | | | | | |

| Table A6 Estimated risk for carotid atherosclerosis in multi-pollutant models according to quantile g-computation. | | | | |
| --- | --- | --- | --- | --- |
| Ambient air pollutant | Coefficient | Weight | Estimate (95% CI) | HR (95% CI) |
| One-year exposure | | | | |
| SO_2_ | 0.463 | 0.667 | 0.412 (0.291-0.533) | 1.510 (1.338-1.704) |
| CO | 0.096 | 0.138 |  |  |
| PM_2.5_ | 0.093 | 0.134 |  |  |
| O_3_ | 0.043 | 0.061 |  |  |
| PM_10_ | -0.205 | 0.724 |  |  |
| NO_2_ | -0.078 | 0.276 |  |  |
| Two-year exposure | | | | |
| SO_2_ | 0.435 | 0.650 | 0.478 (0.356-0.600) | 1.613 (1.428-1.822) |
| CO | 0.099 | 0.147 |  |  |
| PM_2.5_ | 0.092 | 0.137 |  |  |
| O_3_ | 0.044 | 0.066 |  |  |
| PM_10_ | -0.115 | 0.602 |  |  |
| NO_2_ | -0.076 | 0.398 |  |  |
| Covariates in model 3 were adjusted for.  Coefficient indicates the separate effect among the total effect for individual pollutants.  Weight indicates the importance of each pollutant which has the same effect direction.  Estimate indicates the combined estimated coefficient.  PM_2.5_, particulate matter with aerodynamic diameter <2.5 μm; PM_10_, particulate matter with aerodynamic diameter <10 μm; NO_2_, nitrogen dioxide; SO_2_, sulfur dioxide; O_3_, ozone; CO, carbon monoxide; HR, hazard ratio; CI, confidence interval. | | | | |

| Table A7 Sensitivity analyses for the association between PM_2.5_ exposure and carotid atherosclerosis | | | | | | | | | | |  |  |
| --- | --- | --- | --- | --- | --- | --- | --- | --- | --- | --- | --- | --- |
|  | Continuous form | | Categorical form: HR (95% CI: Lower-Upper) | | | | | | | |  |  |
|  | HR (95% CI:  Lower-Upper) | *P* value | Q1 | *P* value | Q2 (95% CI:  Lower-Upper) | *P* value | Q3 (95% CI:  Lower-Upper) | *P* value | Q4 (95% CI:  Lower-Upper) | *P* value | | |
| Simple estimation of individual exposure ^a^ (n=3069) |  |  |  |  |  |  |  |  |  |  | | |
| 1-year exposure | 1.220 (1.128-1.320) | <0.0001 | Ref |  | 1.364 (1.109-1.678) | 0.0032 | 1.380 (1.124-1.694) | 0.0021 | 1.754 (1.435-2.144) | <0.0001 | | |
| 2-year exposure | 1.282 (1.189-1.381) | <0.0001 | Ref |  | 1.398 (1.132-1.726) | 0.0019 | 1.566 (1.273-1.928) | <0.0001 | 2.060 (1.682-2.523) | <0.0001 | | |
| Individual exposure based on workplace address only ^b^ (n=11843) |  |  |  |  |  |  |  |  |  |  | | |
| 1-year exposure | 1.024 (0.991-1.058) | 0.1582 | Ref |  | 1.153 (1.042-1.274) | 0.0058 | 1.124 (1.015-1.245) | 0.0244 | 1.042 (0.938-1.157) | 0.4432 | | |
| 2-year exposure | 1.057 (1.026-1.089) | 0.0003 | Ref |  | 1.114 (1.006-1.234) | 0.0389 | 1.053 (0.950-1.168) | 0.3244 | 1.391 (1.256-1.540) | <0.0001 | | |
| Individuals without medication history at baseline (n=2606) |  |  |  |  |  |  |  |  |  |  | | |
| 1-year exposure | 1.263 (1.150-1.386) | <0.0001 | Ref |  | 1.159 (0.910-1.475) | 0.2327 | 1.464 (1.162-1.846) | 0.0012 | 1.614 (1.281-2.035) | <0.0001 | | |
| 2-year exposure | 1.320 (1.206-1.445) | <0.0001 | Ref |  | 1.284 (1.006-1.639) | 0.0444 | 1.592 (1.259-2.012) | 0.0001 | 1.937 (1.530-2.453) | <0.0001 | | |
| Different years of exposure |  |  |  |  |  |  |  |  |  |  | | |
| 3-year exposure (n=2613) | 1.268 (1.158, 1.388) | <0.0001 | Ref |  | 1.070 (0.834, 1.372) | 0.5937 | 1.273 (1.001, 1.619) | 0.0494 | 1.732 (1.369, 2.190) | <0.0001 | | |
| 4-year exposure (n=2181) | 1.147 (1.027, 1.281) | 0.0152 | Ref |  | 0.918 (0.672, 1.254) | 0.5907 | 1.214 (0.904, 1.629) | 0.1969 | 1.408 (1.053, 1.881) | 0.0209 | | |
| 5-year exposure (n=1802) | 1.133 (0.984, 1.304) | 0.0826 | Ref |  | 0.931 (0.637, 1.360) | 0.7108 | 1.211 (0.848, 1.728) | 0.2920 | 1.305 (0.914, 1.862) | 0.1426 | | |
| Excluding individuals with missing ^c^ values (n=836) |  |  |  |  |  |  |  |  |  |  | | |
| 1-year exposure | 1.228 (1.065, 1.416) | 0.0048 | Ref |  | 0.691 (0.426, 1.121) | 0.1344 | 1.300 (0.854, 1.980) | 0.2204 | 1.460 (0.962, 2.215) | 0.0753 | | |
| 2-year exposure | 1.198 (1.036, 1.384) | 0.0146 | Ref |  | 0.923 (0.578, 1.475) | 0.7389 | 1.340 (0.870, 2.064) | 0.1839 | 1.689 (1.106, 2.580) | 0.0153 | | |
| Two-pollutant model (n=3069) |  |  |  |  |  |  |  |  |  |  | | |
| Plus NO_2_ |  |  |  |  |  |  |  |  |  |  | | |
| 1-year exposure | 1.263 (1.161-1.373) | <0.0001 | Ref |  | 1.184 (0.953-1.472) | 0.1276 | 1.411 (1.140-1.746) | 0.0016 | 1.619 (1.312-1.998) | <0.0001 | | |
| 2-year exposure | 1.322 (1.216-1.436) | <0.0001 | Ref |  | 1.216 (0.978-1.512) | 0.0785 | 1.437 (1.163-1.776) | 0.0008 | 1.803 (1.462-2.222) | <0.0001 | | |
| Plus SO_2_ |  |  |  |  |  |  |  |  |  |  | | |
| 1-year exposure | 1.008 (0.909-1.119) | 0.8773 | Ref |  | 1.074 (0.862-1.339) | 0.5233 | 1.100 (0.879-1.377) | 0.4059 | 1.067 (0.844-1.348) | 0.5877 | | |
| 2-year exposure | 1.079 (0.974-1.194) | 0.1452 | Ref |  | 1.083 (0.868-1.350) | 0.4802 | 1.102 (0.880-1.379) | 0.3979 | 1.165 (0.921-1.474) | 0.2018 | | |
| Plus O_3_ |  |  |  |  |  |  |  |  |  |  | | |
| 1-year exposure | 1.259 (1.158-1.367) | <0.0001 | Ref |  | 1.240 (1.004-1.531) | 0.0453 | 1.463 (1.192-1.797) | 0.0003 | 1.645 (1.342-2.016) | <0.0001 | | |
| 2-year exposure | 1.321 (1.217-1.433) | <0.0001 | Ref |  | 1.324 (1.070-1.639) | 0.0099 | 1.558 (1.269-1.913) | <0.0001 | 1.923 (1.565-2.363) | <0.0001 | | |
| Plus CO |  |  |  |  |  |  |  |  |  |  | | |
| 1-year exposure | 1.254 (1.151-1.365) | <0.0001 | Ref |  | 1.111 (0.896-1.379) | 0.3374 | 1.310 (1.060-1.619) | 0.0126 | 1.522 (1.236-1.874) | <0.0001 | | |
| 2-year exposure | 1.325 (1.217-1.442) | <0.0001 | Ref |  | 1.147 (0.924-1.424) | 0.2130 | 1.362 (1.105-1.680) | 0.0038 | 1.692 (1.374-2.085) | <0.0001 | |  |
| Variables was adjusted for in model 3.  - Because of high correlation and collinearity, two-pollutant model was not conducted.  ^a^ The estimation of individual exposure to pollutants was based on personal activity patterns, but did not consider the infiltration from outdoor to indoor environment.  ^b^ Individuals with complete work address (n=11843) were included in the analysis.  ^c^ Individuals with missing values of time-weighted variables were excluded (n=836).  Ref, Reference; PM_2.5_, particulate matter with aerodynamic diameter <2.5 μm; Q, quartile; HR, hazard ratio; CI, confidence interval; NO_2_, nitrogen dioxide; SO_2_, sulfur dioxide; O_3_, ozone; CO, carbon monoxide. | | | | | | | | | | |  |  |

| Table A8 Sensitivity analyses for the association between PM_10_ exposure and carotid atherosclerosis | | | | | | | | | | |  |
| --- | --- | --- | --- | --- | --- | --- | --- | --- | --- | --- | --- |
|  | Continuous form | | Categorical form: HR (95% CI: Lower-Upper) | | | | | | | |  |
|  | HR (95% CI:  Lower-Upper) | *P* value | Q1 | *P* value | Q2 (95% CI:  Lower-Upper) | *P* value | Q3 (95% CI:  Lower-Upper) | *P* value | Q4 (95% CI:  Lower-Upper) | *P* value | |
| Simple estimation of individual exposure ^a^ (n=3069) |  |  |  |  |  |  |  |  |  |  | |
| 1-year exposure | 1.156 (1.064-1.257) | 0.0007 | Ref |  | 1.841 (1.488-2.279) | <0.0001 | 1.641 (1.327-2.029) | <0.0001 | 1.283 (1.030-1.598) | 0.0260 | |
| 2-year exposure | 1.239 (1.139-1.348) | <0.0001 | Ref |  | 1.454 (1.171-1.805) | 0.0007 | 1.779 (1.440-2.198) | <0.0001 | 1.487 (1.198-1.847) | 0.0003 | |
| Individual exposure based on workplace address only ^b^ (n=11843) |  |  |  |  |  |  |  |  |  |  | |
| 1-year exposure | 1.024 (0.991-1.058) | 0.1582 | Ref |  | 1.152 (1.042-1.274) | 0.0058 | 1.124 (1.015-1.245) | 0.0244 | 1.042 (0.938-1.157) | 0.4432 | |
| 2-year exposure | 1.057 (1.026-1.089) | 0.0003 | Ref |  | 1.114 (1.006-1.234) | 0.0389 | 1.053 (0.950-1.168) | 0.3244 | 1.391 (1.256-1.540) | <0.0001 | |
| Individuals without medication history at baseline (n=2606) |  |  |  |  |  |  |  |  |  |  | |
| 1-year exposure | 1.190 (1.080-1.310) | 0.0004 | Ref |  | 1.350 (1.054-1.728) | 0.0173 | 1.487 (1.166-1.896) | 0.0014 | 1.409 (1.107-1.793) | 0.0053 | |
| 2-year exposure | 1.248 (1.133-1.375) | <0.0001 | Ref |  | 1.158 (0.902-1.487) | 0.2493 | 1.582 (1.246-2.008) | 0.0002 | 1.535 (1.208-1.949) | 0.0004 | |
| Different years of exposure |  |  |  |  |  |  |  |  |  |  | |
| 3-year exposure (n=2613) | 1.179 (1.066, 1.304) | 0.0013 | Ref |  | 1.001 (0.778, 1.288) | 0.9964 | 1.329 (1.045, 1.691) | 0.0205 | 1.284 (1.006, 1.639) | 0.0445 | |
| 4-year exposure (n=2181) | 1.166 (1.022, 1.331) | 0.0222 | Ref |  | 1.131 (0.820, 1.559) | 0.4538 | 1.520 (1.120, 2.063) | 0.0071 | 1.352 (0.984, 1.856) | 0.0626 | |
| 5-year exposure (n=1802) | 1.188 (0.994, 1.419) | 0.0586 | Ref |  | 0.946 (0.627, 1.428) | 0.7926 | 1.610 (1.106, 2.344) | 0.0130 | 1.274 (0.860, 1.889) | 0.2271 | |
| Excluding individuals with missing ^c^ values (n=836) |  |  |  |  |  |  |  |  |  |  | |
| 1-year exposure | 1.424 (1.134, 1.786) | 0.0023 | Ref |  | 1.215 (0.756, 1.954) | 0.4214 | 1.262 (0.804, 1.984) | 0.3120 | 1.731 (1.097, 2.732) | 0.0184 | |
| 2-year exposure | 1.356 (1.084, 1.697) | 0.0078 | Ref |  | 1.215 (0.756, 1.953) | 0.4203 | 1.117 (0.714, 1.748) | 0.6272 | 1.815 (1.162, 2.836) | 0.0088 | |
| Two-pollutant model (n=3069) |  |  |  |  |  |  |  |  |  |  | |
| Plus NO_2_ |  |  |  |  |  |  |  |  |  |  | |
| 1-year exposure | - | - |  |  | - | - | - | - | - | - | |
| 2-year exposure | 1.205 (1.090-1.332) | 0.0003 | Ref |  | 1.096 (0.861-1.395) | 0.4576 | 1.424 (1.114-1.820) | 0.0048 | 1.360 (1.055-1.754) | 0.0178 | |
| Plus O_3_ |  |  |  |  |  |  |  |  |  |  | |
| 1-year exposure | 1.147 (1.055-1.247) | 0.0013 | Ref |  | 1.300 (1.045-1.616) | 0.0186 | 1.433 (1.155-1.778) | 0.0011 | 1.292 (1.049-1.591) | 0.0161 | |
| 2-year exposure | 1.201 (1.104-1.306) | <0.0001 | Ref |  | 1.270 (1.016-1.587) | 0.0356 | 1.686 (1.361-2.088) | <0.0001 | 1.500 (1.216-1.850) | 0.0002 | |
| Plus CO |  |  |  |  |  |  |  |  |  |  | |
| 1-year exposure | 1.102 (1.001-1.214) | 0.0479 | Ref |  | 1.032 (0.811-1.314) | 0.7966 | 1.078 (0.842-1.380) | 0.5528 | 1.006 (0.785-1.288) | 0.9637 | |
| 2-year exposure | 1.135 (1.032-1.248) | 0.0088 | Ref |  | 0.977 (0.771-1.237) | 0.8466 | 1.248 (0.987-1.579) | 0.0639 | 1.155 (0.912-1.462) | 0.2311 | |
| Variables was adjusted for in model 3.  - Because of high correlation and collinearity, two-pollutant model was not conducted.  ^a^ The estimation of individual exposure to pollutants was based on personal activity patterns, but did not consider the infiltration from outdoor to indoor environment.  ^b^ Individuals with complete work address (n=11843) were included in the analysis.  ^c^ Individuals with missing values of time-weighted variables were excluded (n=836).  Ref, Reference; PM_2.5_, particulate matter with aerodynamic diameter <2.5 μm; Q, quartile; HR, hazard ratio; CI, confidence interval; NO_2_, nitrogen dioxide; SO_2_, sulfur dioxide; O_3_, ozone; CO, carbon monoxide. | | | | | | | | | | |  |

| \| Table A9 Subgroup analyses exploring the association between individual PM_2.5_ exposure and carotid atherosclerosis \| \| \| \| \| \| \| \| \| \| \| \| \| --- \| --- \| --- \| --- \| --- \| --- \| --- \| --- \| --- \| --- \| --- \| --- \| \| Exposure \| Subgroups  (Number) \| Continuous form \| \| Categorical form: HR (95% CI: Lower-Upper) \| \| \| \| \| \| \| \| \| HR (95% CI:  Lower-Upper) \| P value \| Q1 \| P value \| Q2 (95% CI:  Lower-Upper) \| P value \| Q3 (95% CI:  Lower-Upper) \| P value \| Q4 (95% CI:  Lower-Upper) \| P value \| \|  \| Age (years) \|  \|  \|  \|  \|  \|  \|  \|  \|  \|  \| \| 1-year \| <60 (n=2872) \| 1.286 (1.179-1.402) \| <0.0001 \| Ref \|  \| 1.266 (1.010-1.587) \| 0.0406 \| 1.471 (1.181-1.833) \| 0.0006 \| 1.769 (1.424-2.196) \| <0.0001 \| \| ≥60 (n=197) \| 1.016 (0.808-1.277) \| 0.8948 \| Ref \|  \| 0.827 (0.486-1.408) \| 0.4843 \| 1.283 (0.745-2.210) \| 0.3682 \| 0.960 (0.571-1.615) \| 0.8779 \| \| 2-year \| <60 (n=2872) \| 1.346 (1.236-1.465) \| <0.0001 \| Ref \|  \| 1.334 (1.062-1.675) \| 0.0131 \| 1.572 (1.260-1.961) \| <0.0001 \| 2.064 (1.658-2.570) \| <0.0001 \| \| ≥60 (n=197) \| 1.073 (0.859-1.342) \| 0.5347 \| Ref \|  \| 1.043 (0.625-1.741) \| 0.8718 \| 1.158 (0.668-2.007) \| 0.6020 \| 1.425 (0.845-2.404) \| 0.1842 \| \|  \| Sex \|  \|  \|  \|  \|  \|  \|  \|  \|  \|  \| \| 1-year \| Male (n=1420) \| 1.376 (1.236-1.531) \| <0.0001 \| Ref \|  \| 1.127 (0.854-1.486) \| 0.3979 \| 1.638 (1.264-2.122) \| 0.0002 \| 1.824 (1.409-2.363) \| <0.0001 \| \| Female (n=1649) \| 1.141 (1.002-1.299) \| 0.0457 \| Ref \|  \| 1.376 (0.992-1.910) \| 0.0562 \| 1.189 (0.849-1.666) \| 0.3136 \| 1.482 (1.064-2.065) \| 0.0199 \| \| 2-year \| Male (n=1420) \| 1.441 (1.297-1.602) \| <0.0001 \| Ref \|  \| 1.304 (0.983-1.730) \| 0.0659 \| 1.778 (1.364-2.317) \| <0.0001 \| 2.277 (1.748-2.967) \| <0.0001 \| \| Female (n=1649) \| 1.199 (1.056-1.361) \| 0.0052 \| Ref \|  \| 1.215 (0.876-1.685) \| 0.2436 \| 1.256 (0.902-1.748) \| 0.1769 \| 1.433 (1.030-1.994) \| 0.0328 \| \|  \| BMI (kg/m2) \|  \|  \|  \|  \|  \|  \|  \|  \|  \|  \| \| 1-year \| ≥24 (n=1445) \| 1.347 (1.215-1.493) \| <0.0001 \| Ref \|  \| 1.138 (0.869-1.490) \| 0.3482 \| 1.530 (1.188-1.971) \| 0.0010 \| 1.808 (1.404-2.328) \| <0.0001 \| \| <24 (n=1624) \| 1.156 (1.004-1.330) \| 0.0433 \| Ref \|  \| 1.478 (1.053-2.073) \| 0.0239 \| 1.316 (0.930-1.862) \| 0.1204 \| 1.575 (1.120-2.214) \| 0.0090 \| \| 2-year \| ≥24 (n=1445) \| 1.428 (1.286-1.585) \| <0.0001 \| Ref \|  \| 1.225 (0.934-1.606) \| 0.1433 \| 1.713 (1.327-2.211) \| <0.0001 \| 2.033 (1.571-2.632) \| <0.0001 \| \| <24 (n=1624) \| 1.191 (1.042-1.362) \| 0.0105 \| Ref \|  \| 1.392 (0.993-1.951) \| 0.0551 \| 1.313 (0.934-1.847) \| 0.1167 \| 1.716 (1.223-2.409) \| 0.0018 \| \|  \| Hyperuricemia \|  \|  \|  \|  \|  \|  \|  \|  \|  \|  \| \| 1-year \| Yes (n=357) \| 1.498 (1.216-1.845) \| 0.0001 \| Ref \|  \| 1.709 (0.926-3.151) \| 0.0864 \| 2.142 (1.195-3.838) \| 0.0105 \| 2.515 (1.393-4.542) \| 0.0022 \| \| No (n=2712) \| 1.224 (1.119-1.340) \| <0.0001 \| Ref \|  \| 1.225 (0.979-1.533) \| 0.0756 \| 1.363 (1.096-1.697) \| 0.0055 \| 1.614 (1.299-2.005) \| <0.0001 \| \| 2-year \| Yes (n=357) \| 1.509 (1.231-1.851) \| <0.0001 \| Ref \|  \| 2.031 (1.096-3.764) \| 0.0243 \| 2.083 (1.125-3.853) \| 0.0195 \| 3.086 (1.681-5.666) \| 0.0003 \| \| No (n=2712) \| 1.297 (1.187-1.417) \| <0.0001 \| Ref \|  \| 1.247 (0.996-1.562) \| 0.0547 \| 1.462 (1.176-1.817) \| 0.0006 \| 1.813 (1.458-2.254) \| <0.0001 \| \|  \| TG (mmol/L) \|  \|  \|  \|  \|  \|  \|  \|  \|  \|  \| \| 1-year \| ≥1.7 (n=858) \| 1.256 (1.088-1.450) \| 0.0019 \| Ref \|  \| 1.238 (0.852-1.800) \| 0.2622 \| 1.368 (0.951-1.969) \| 0.0912 \| 1.675 (1.164-2.410) \| 0.0054 \| \| <1.7 (n=2211) \| 1.277 (1.155-1.413) \| <0.0001 \| Ref \|  \| 1.216 (0.942-1.571) \| 0.1337 \| 1.508 (1.178-1.930) \| 0.0011 \| 1.751 (1.372-2.235) \| <0.0001 \| \| 2-year \| ≥1.7 (n=858) \| 1.333 (1.152-1.542) \| 0.0001 \| Ref \|  \| 1.483 (1.010-2.176) \| 0.0443 \| 1.798 (1.252-2.583) \| 0.0015 \| 1.918 (1.322-2.785) \| 0.0006 \| \| <1.7 (n=2211) \| 1.330 (1.208-1.464) \| <0.0001 \| Ref \|  \| 1.261 (0.977-1.628) \| 0.0746 \| 1.494 (1.164-1.918) \| 0.0016 \| 2.030 (1.586-2.599) \| <0.0001 \| \|  \| LDL-C (mmol/L) \|  \|  \|  \|  \|  \|  \|  \|  \|  \|  \| \| 1-year \| ≥3.4 (n=558) \| 1.368 (1.173-1.596) \| <0.0001 \| Ref \|  \| 1.692 (1.148-2.496) \| 0.0080 \| 1.886 (1.263-2.815) \| 0.0019 \| 2.102 (1.417-3.118) \| 0.0002 \| \| <3.4 (n=2511) \| 1.232 (1.116-1.359) \| <0.0001 \| Ref \|  \| 1.075 (0.838-1.378) \| 0.5688 \| 1.285 (1.011-1.633) \| 0.0404 \| 1.583 (1.252-2.001) \| 0.0001 \| \| 2-year \| ≥3.4 (n=558) \| 1.427 (1.224-1.664) \| <0.0001 \| Ref \|  \| 1.388 (0.933-2.064) \| 0.1058 \| 2.021 (1.373-2.976) \| 0.0004 \| 2.164 (1.461-3.206) \| 0.0001 \| \| <3.4 (n=2511) \| 1.303 (1.183-1.435) \| <0.0001 \| Ref \|  \| 1.200 (0.936-1.540) \| 0.1510 \| 1.397 (1.097-1.779) \| 0.0067 \| 1.845 (1.452-2.344) \| <0.0001 \| \|  \| HDL-C (mmol/L) \|  \|  \|  \|  \|  \|  \|  \|  \|  \|  \| \| 1-year \| <1.0 (n=403) \| 1.038 (0.803-1.343) \| 0.7745 \| Ref \|  \| 0.534 (0.293-0.976) \| 0.0416 \| 1.023 (0.593-1.764) \| 0.9355 \| 0.871 (0.506-1.501) \| 0.6201 \| \| ≥1.0 (n=2666) \| 1.298 (1.188-1.417) \| <0.0001 \| Ref \|  \| 1.355 (1.080-1.699) \| 0.0087 \| 1.519 (1.218-1.895) \| 0.0002 \| 1.792 (1.438-2.233) \| <0.0001 \| \| 2-year \| <1.0 (n=403) \| 1.101 (0.858-1.415) \| 0.4494 \| Ref \|  \| 0.777 (0.424-1.424) \| 0.4141 \| 1.299 (0.738-2.286) \| 0.3650 \| 1.213 (0.690-2.131) \| 0.5028 \| \| ≥1.0 (n=2666) \| 1.358 (1.245-1.480) \| <0.0001 \| Ref \|  \| 1.417 (1.128-1.780) \| 0.0028 \| 1.584 (1.269-1.978) \| <0.0001 \| 2.043 (1.636-2.552) \| <0.0001 \| \| Variables was adjusted for in model 3.  Ref, Reference; PM_2.5_, particulate matter with aerodynamic diameter <2.5 μm; Q, quartile; HR, hazard ratio; CI, confidence interval; BMI, body mass index; LDL-C, low-density lipoprotein cholesterol; TG, triglycerides; HDL-C, high-density lipoprotein cholesterol. \| \| \| \| \| \| \| \| \| \| \| \| |
| --- | --- | --- | --- | --- | --- | --- | --- | --- | --- | --- | --- | --- | --- | --- | --- | --- | --- | --- | --- | --- | --- | --- | --- | --- | --- | --- | --- | --- | --- | --- | --- | --- | --- | --- | --- | --- | --- | --- | --- | --- | --- | --- | --- | --- | --- | --- | --- | --- | --- | --- | --- | --- | --- | --- | --- | --- | --- | --- | --- | --- | --- | --- | --- | --- | --- | --- | --- | --- | --- | --- | --- | --- | --- | --- | --- | --- | --- | --- | --- | --- | --- | --- | --- | --- | --- | --- | --- | --- | --- | --- | --- | --- | --- | --- | --- | --- | --- | --- | --- | --- | --- | --- | --- | --- | --- | --- | --- | --- | --- | --- | --- | --- | --- | --- | --- | --- | --- | --- | --- | --- | --- | --- | --- | --- | --- | --- | --- | --- | --- | --- | --- | --- | --- | --- | --- | --- | --- | --- | --- | --- | --- | --- | --- | --- | --- | --- | --- | --- | --- | --- | --- | --- | --- | --- | --- | --- | --- | --- | --- | --- | --- | --- | --- | --- | --- | --- | --- | --- | --- | --- | --- | --- | --- | --- | --- | --- | --- | --- | --- | --- | --- | --- | --- | --- | --- | --- | --- | --- | --- | --- | --- | --- | --- | --- | --- | --- | --- | --- | --- | --- | --- | --- | --- | --- | --- | --- | --- | --- | --- | --- | --- | --- | --- | --- | --- | --- | --- | --- | --- | --- | --- | --- | --- | --- | --- | --- | --- | --- | --- | --- | --- | --- | --- | --- | --- | --- | --- | --- | --- | --- | --- | --- | --- | --- | --- | --- | --- | --- | --- | --- | --- | --- | --- | --- | --- | --- | --- | --- | --- | --- | --- | --- | --- | --- | --- | --- | --- | --- | --- | --- | --- | --- | --- | --- | --- | --- | --- | --- | --- | --- | --- | --- | --- | --- | --- | --- | --- | --- | --- | --- | --- | --- | --- | --- | --- | --- | --- | --- | --- | --- | --- | --- | --- | --- | --- | --- | --- | --- | --- | --- | --- | --- | --- | --- | --- | --- | --- | --- | --- | --- | --- | --- | --- | --- | --- | --- | --- | --- | --- | --- | --- | --- | --- | --- | --- | --- | --- | --- | --- | --- | --- | --- | --- | --- | --- | --- | --- | --- | --- | --- | --- | --- | --- | --- | --- | --- | --- | --- | --- | --- | --- | --- | --- | --- | --- | --- | --- | --- | --- | --- | --- | --- | --- | --- | --- | --- | --- | --- | --- | --- | --- | --- | --- | --- | --- | --- | --- | --- | --- | --- | --- | --- | --- | --- | --- | --- | --- | --- | --- | --- | --- | --- | --- | --- | --- | --- | --- | --- | --- | --- | --- | --- | --- | --- | --- | --- | --- | --- | --- | --- | --- | --- | --- | --- | --- | --- | --- | --- | --- | --- | --- | --- | --- | --- | --- | --- | --- | --- | --- | --- | --- | --- | --- | --- | --- | --- | --- | --- | --- | --- | --- | --- |

| \| Table A10 Subgroup analyses exploring the association between individual PM_10_ exposure and carotid atherosclerosis \| \| \| \| \| \| \| \| \| \| \| \| \| --- \| --- \| --- \| --- \| --- \| --- \| --- \| --- \| --- \| --- \| --- \| --- \| \| Exposure \| Subgroups  (Number) \| Continuous form \| \| Categorical form: HR (95% CI: Lower-Upper) \| \| \| \| \| \| \| \| \| HR (95% CI:  Lower-Upper) \| *P* value \| Q1 \| *P* value \| Q2 (95% CI:  Lower-Upper) \| *P* value \| Q3 (95% CI:  Lower-Upper) \| *P* value \| Q4 (95% CI:  Lower-Upper) \| *P* value \| \|  \| Age (years) \|  \|  \|  \|  \|  \|  \|  \|  \|  \|  \| \| 1-year \| <60 (n=2872) \| 1.180 (1.079-1.290) \| 0.0003 \| Ref \|  \| 1.306 (1.040-1.639) \| 0.0214 \| 1.521 (1.219-1.898) \| 0.0002 \| 1.379 (1.103-1.723) \| 0.0048 \| \| ≥60 (n=197) \| 0.940 (0.697-1.267) \| 0.6849 \| Ref \|  \| 0.954 (0.531-1.715) \| 0.8747 \| 0.693 (0.389-1.236) \| 0.2142 \| 1.243 (0.715-2.164) \| 0.4409 \| \| 2-year \| <60 (n=2872) \| 1.236 (1.129-1.352) \| <0.0001 \| Ref \|  \| 1.150 (0.915-1.444) \| 0.2316 \| 1.539 (1.237-1.914) \| 0.0001 \| 1.492 (1.198-1.859) \| 0.0004 \| \| ≥60 (n=197) \| 0.996 (0.732-1.356) \| 0.9813 \| Ref \|  \| 1.000 (0.567-1.767) \| 0.9987 \| 0.652 (0.352-1.205) \| 0.1721 \| 1.208 (0.689-2.117) \| 0.5100 \| \|  \| Sex \|  \|  \|  \|  \|  \|  \|  \|  \|  \|  \| \| 1-year \| Male (n=1420) \| 1.128 (1.015-1.253) \| 0.0253 \| Ref \|  \| 1.063 (0.814-1.388) \| 0.6530 \| 1.357 (1.050-1.755) \| 0.0199 \| 1.162 (0.893-1.512) \| 0.2637 \| \| Female (n=1649) \| 1.214 (1.064-1.384) \| 0.0039 \| Ref \|  \| 1.291 (0.907-1.838) \| 0.1564 \| 1.368 (0.964-1.940) \| 0.0791 \| 1.363 (0.969-1.918) \| 0.0752 \| \| 2-year \| Male (n=1420) \| 1.190 (1.069-1.324) \| 0.0014 \| Ref \|  \| 1.163 (0.889-1.520) \| 0.2705 \| 1.613 (1.245-2.090) \| 0.0003 \| 1.428 (1.098-1.858) \| 0.0080 \| \| Female (n=1649) \| 1.256 (1.100-1.435) \| 0.0007 \| Ref \|  \| 1.103 (0.776-1.569) \| 0.5843 \| 1.376 (0.980-1.933) \| 0.0657 \| 1.359 (0.974-1.898) \| 0.0714 \| \|  \| BMI (kg/m2) \|  \|  \|  \|  \|  \|  \|  \|  \|  \|  \| \| 1-year \| ≥24 (n=1445) \| 1.169 (1.052-1.298) \| 0.0036 \| Ref \|  \| 1.254 (0.965-1.629) \| 0.0906 \| 1.231 (0.947-1.600) \| 0.1211 \| 1.288 (0.995-1.667) \| 0.0544 \| \| <24 (n=1624) \| 1.105 (0.964-1.268) \| 0.1526 \| Ref \|  \| 1.007 (0.702-1.444) \| 0.9704 \| 1.397 (0.996-1.960) \| 0.0525 \| 1.127 (0.800-1.588) \| 0.4942 \| \| 2-year \| ≥24 (n=1445) \| 1.233 (1.106-1.375) \| 0.0002 \| Ref \|  \| 1.338 (1.030-1.738) \| 0.0294 \| 1.454 (1.120-1.887) \| 0.0049 \| 1.505 (1.161-1.951) \| 0.0020 \| \| <24 (n=1624) \| 1.157 (1.011-1.323) \| 0.0338 \| Ref \|  \| 0.818 (0.570-1.172) \| 0.2726 \| 1.420 (1.021-1.975) \| 0.0371 \| 1.228 (0.880-1.714) \| 0.2266 \| \|  \| Hyperuricemia \|  \|  \|  \|  \|  \|  \|  \|  \|  \|  \| \| 1-year \| Yes (n=357) \| 1.223 (0.968-1.546) \| 0.0919 \| Ref \|  \| 2.571 (1.412-4.681) \| 0.0020 \| 2.051 (1.111-3.788) \| 0.0217 \| 1.767 (0.929-3.358) \| 0.0825 \| \| No (n=2712) \| 1.143 (1.045-1.251) \| 0.0035 \| Ref \|  \| 1.181 (0.940-1.484) \| 0.1533 \| 1.375 (1.099-1.719) \| 0.0053 \| 1.262 (1.011-1.576) \| 0.0401 \| \| 2-year \| Yes (n=357) \| 1.293 (1.029-1.626) \| 0.0276 \| Ref \|  \| 2.489 (1.376-4.501) \| 0.0026 \| 1.877 (1.019-3.456) \| 0.0433 \| 1.809 (0.968-3.381) \| 0.0633 \| \| No (n=2712) \| 1.201 (1.098-1.313) \| <0.0001 \| Ref \|  \| 1.073 (0.852-1.351) \| 0.5521 \| 1.525 (1.226-1.897) \| 0.0002 \| 1.420 (1.140-1.770) \| 0.0018 \| \|  \| TG (mmol/L) \|  \|  \|  \|  \|  \|  \|  \|  \|  \|  \| \| 1-year \| ≥1.7 (n=858) \| 1.209 (1.043-1.401) \| 0.0117 \| Ref \|  \| 1.156 (0.780-1.713) \| 0.4712 \| 1.526 (1.043-2.231) \| 0.0294 \| 1.243 (0.831-1.860) \| 0.2895 \| \| <1.7 (n=2211) \| 1.134 (1.024-1.256) \| 0.0161 \| Ref \|  \| 1.207 (0.932-1.561) \| 0.1534 \| 1.298 (1.007-1.673) \| 0.0442 \| 1.243 (0.968-1.595) \| 0.0879 \| \| 2-year \| ≥1.7 (n=858) \| 1.299 (1.116-1.511) \| 0.0007 \| Ref \|  \| 1.172 (0.802-1.713) \| 0.4113 \| 1.526 (1.056-2.205) \| 0.0246 \| 1.686 (1.155-2.459) \| 0.0067 \| \| <1.7 (n=2211) \| 1.186 (1.070-1.314) \| 0.0011 \| Ref \|  \| 1.106 (0.855-1.431) \| 0.4414 \| 1.439 (1.124-1.842) \| 0.0038 \| 1.358 (1.061-1.739) \| 0.0153 \| \|  \| LDL-C (mmol/L) \|  \|  \|  \|  \|  \|  \|  \|  \|  \|  \| \| 1-year \| ≥3.4 (n=558) \| 1.276 (1.102-1.477) \| 0.0011 \| Ref \|  \| 1.603 (1.096-2.345) \| 0.0149 \| 1.738 (1.202-2.513) \| 0.0033 \| 1.522 (1.041-2.224) \| 0.0302 \| \| <3.4 (n=2511) \| 1.123 (1.015-1.244) \| 0.0250 \| Ref \|  \| 1.214 (0.942-1.563) \| 0.1335 \| 1.309 (1.016-1.686) \| 0.0369 \| 1.260 (0.981-1.617) \| 0.0699 \| \| 2-year \| ≥3.4 (n=558) \| 1.307 (1.129-1.513) \| 0.0003 \| Ref \|  \| 1.319 (0.898-1.938) \| 0.1583 \| 1.812 (1.259-2.607) \| 0.0014 \| 1.556 (1.072-2.260) \| 0.0202 \| \| <3.4 (n=2511) \| 1.189 (1.073-1.317) \| 0.0010 \| Ref \|  \| 1.102 (0.854-1.421) \| 0.4570 \| 1.470 (1.148-1.882) \| 0.0022 \| 1.377 (1.075-1.764) \| 0.0113 \| \|  \| HDL-C (mmol/L) \|  \|  \|  \|  \|  \|  \|  \|  \|  \|  \| \| 1-year \| <1.0 (n=403) \| 1.089 (0.819-1.448) \| 0.5580 \| Ref \|  \| 1.252 (0.680-2.306) \| 0.4704 \| 1.583 (0.844-2.968) \| 0.1521 \| 1.546 (0.825-2.897) \| 0.1741 \| \| ≥1.0 (n=2666) \| 1.147 (1.049-1.253) \| 0.0025 \| Ref \|  \| 1.212 (0.965-1.521) \| 0.0986 \| 1.391 (1.113-1.739) \| 0.0037 \| 1.242 (0.993-1.554) \| 0.0571 \| \| 2-year \| <1.0 (n=403) \| 1.160 (0.868-1.549) \| 0.3159 \| Ref \|  \| 1.145 (0.630-2.080) \| 0.6566 \| 1.187 (0.640-2.202) \| 0.5871 \| 1.435 (0.785-2.623) \| 0.2405 \| \| ≥1.0 (n=2666) \| 1.200 (1.097-1.312) \| <0.0001 \| Ref \|  \| 1.235 (0.981-1.554) \| 0.0726 \| 1.591 (1.274-1.986) \| <0.0001 \| 1.420 (1.134-1.779) \| 0.0023 \| \| Variables was adjusted for in model 3.  Ref, Reference; PM_10_, particulate matter with aerodynamic diameter <10 μm; Q, quartile; HR, hazard ratio; CI, confidence interval; BMI, body mass index; LDL-C, low-density lipoprotein cholesterol; TG, triglycerides; HDL-C, high-density lipoprotein cholesterol. \| \| \| \| \| \| \| \| \| \| \| \| |
| --- | --- | --- | --- | --- | --- | --- | --- | --- | --- | --- | --- | --- | --- | --- | --- | --- | --- | --- | --- | --- | --- | --- | --- | --- | --- | --- | --- | --- | --- | --- | --- | --- | --- | --- | --- | --- | --- | --- | --- | --- | --- | --- | --- | --- | --- | --- | --- | --- | --- | --- | --- | --- | --- | --- | --- | --- | --- | --- | --- | --- | --- | --- | --- | --- | --- | --- | --- | --- | --- | --- | --- | --- | --- | --- | --- | --- | --- | --- | --- | --- | --- | --- | --- | --- | --- | --- | --- | --- | --- | --- | --- | --- | --- | --- | --- | --- | --- | --- | --- | --- | --- | --- | --- | --- | --- | --- | --- | --- | --- | --- | --- | --- | --- | --- | --- | --- | --- | --- | --- | --- | --- | --- | --- | --- | --- | --- | --- | --- | --- | --- | --- | --- | --- | --- | --- | --- | --- | --- | --- | --- | --- | --- | --- | --- | --- | --- | --- | --- | --- | --- | --- | --- | --- | --- | --- | --- | --- | --- | --- | --- | --- | --- | --- | --- | --- | --- | --- | --- | --- | --- | --- | --- | --- | --- | --- | --- | --- | --- | --- | --- | --- | --- | --- | --- | --- | --- | --- | --- | --- | --- | --- | --- | --- | --- | --- | --- | --- | --- | --- | --- | --- | --- | --- | --- | --- | --- | --- | --- | --- | --- | --- | --- | --- | --- | --- | --- | --- | --- | --- | --- | --- | --- | --- | --- | --- | --- | --- | --- | --- | --- | --- | --- | --- | --- | --- | --- | --- | --- | --- | --- | --- | --- | --- | --- | --- | --- | --- | --- | --- | --- | --- | --- | --- | --- | --- | --- | --- | --- | --- | --- | --- | --- | --- | --- | --- | --- | --- | --- | --- | --- | --- | --- | --- | --- | --- | --- | --- | --- | --- | --- | --- | --- | --- | --- | --- | --- | --- | --- | --- | --- | --- | --- | --- | --- | --- | --- | --- | --- | --- | --- | --- | --- | --- | --- | --- | --- | --- | --- | --- | --- | --- | --- | --- | --- | --- | --- | --- | --- | --- | --- | --- | --- | --- | --- | --- | --- | --- | --- | --- | --- | --- | --- | --- | --- | --- | --- | --- | --- | --- | --- | --- | --- | --- | --- | --- | --- | --- | --- | --- | --- | --- | --- | --- | --- | --- | --- | --- | --- | --- | --- | --- | --- | --- | --- | --- | --- | --- | --- | --- | --- | --- | --- | --- | --- | --- | --- | --- | --- | --- | --- | --- | --- | --- | --- | --- | --- | --- | --- | --- | --- | --- | --- | --- | --- | --- | --- | --- | --- | --- | --- | --- | --- | --- | --- | --- | --- | --- | --- | --- | --- | --- | --- | --- | --- | --- | --- | --- | --- | --- | --- | --- | --- | --- | --- | --- | --- | --- | --- | --- | --- | --- | --- | --- | --- | --- | --- | --- | --- | --- | --- | --- | --- | --- | --- | --- | --- | --- | --- | --- | --- | --- | --- |


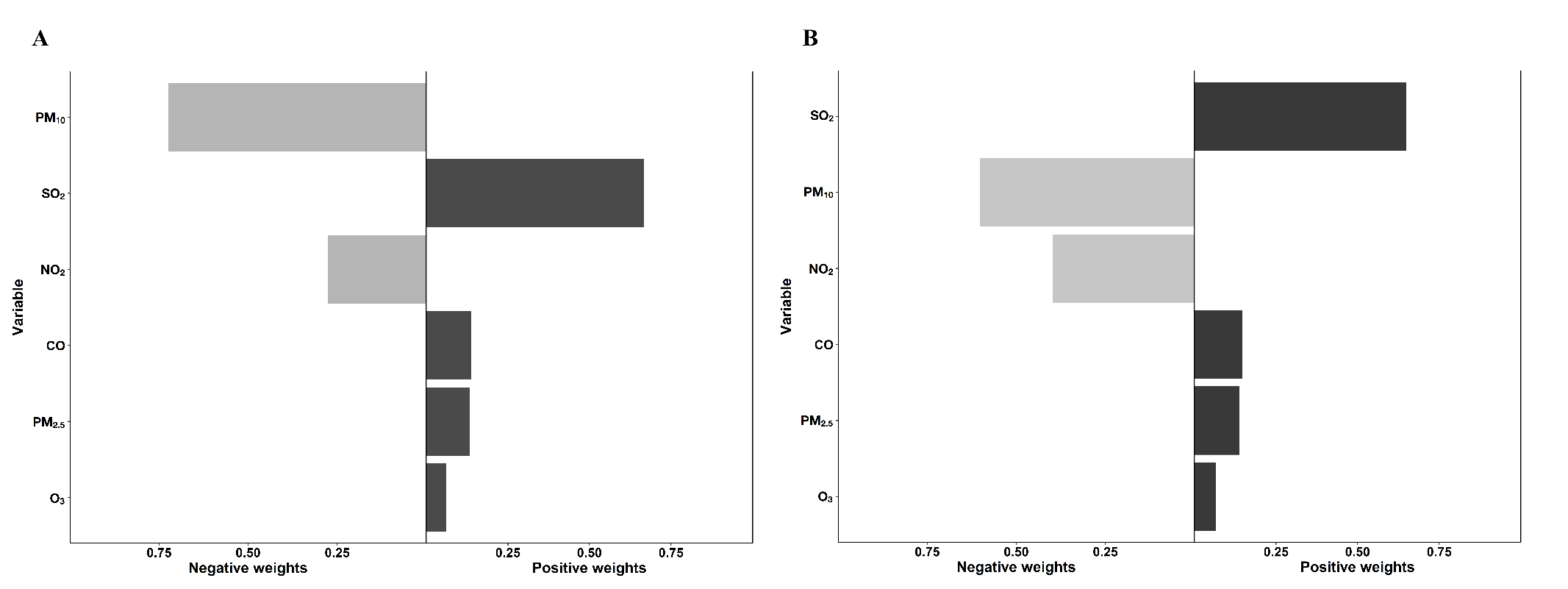


Fig. A1 Combined effect of personal time-weighted average exposure to mixed ambient pollutants on the risk of carotid atherosclerosis.

A and B represented the weight of one- and two-year co-exposure to ambient air pollutants on the risk of carotid atherosclerosis, respectively, according to quantile g-computation regression.

PM_10_, particulate matter with aerodynamic diameter <10 μm; PM_2.5_, particulate matter with aerodynamic diameter <2.5 μm; NO_2_, nitrogen dioxide; SO_2_, sulfur dioxide; O_3_, ozone; CO, carbon monoxide
